# A comprehensive review of *Wolbachia*-mediated mechanisms to control dengue virus transmission in *Aedes aegypti* through innate immune pathways

**DOI:** 10.3389/fimmu.2024.1434003

**Published:** 2024-08-08

**Authors:** Iqra Mushtaq, Muhammad Sajjad Sarwar, Iqra Munzoor

**Affiliations:** Department of Zoology, Faculty of Life Sciences, University of Okara, Okara, Pakistan

**Keywords:** dengue virus, *Aedes aegypti*, *Wolbachia*, innate immune pathways, toll pathway, IMD pathway, JAK/STAT pathway

## Abstract

The Dengue virus (DENV), primarily spread by *Aedes aegypti* and also by *Aedes albopictus* in some regions, poses significant global health risks. Alternative techniques are urgently needed because the current control mechanisms are insufficient to reduce the transmission of DENV. Introducing *Wolbachia pipientis* into *Ae. aegypti* inhibits DENV transmission, however, the underlying mechanisms are still poorly understood. Innate immune effector upregulation, the regulation of autophagy, and intracellular competition between *Wolbachia* and DENV for lipids are among the theories for the mechanism of inhibition. Furthermore, mainly three immune pathways Toll, IMD, and JAK/STAT are involved in the host for the suppression of the virus. These pathways are activated by *Wolbachia* and DENV in the host and are responsible for the upregulation and downregulation of many genes in mosquitoes, which ultimately reduces the titer of the DENV in the host. The functioning of these immune pathways depends upon the *Wolbachia*, host, and virus interaction. Here, we summarize the current understanding of DENV recognition by the *Ae. aegypti*’s immune system, aiming to create a comprehensive picture of our knowledge. Additionally, we investigated how *Wolbachia* regulates the activation of multiple genes associated with immune priming for the reduction of DENV.

## 1 Introduction

Arboviruses are mainly transmitted by blood-feeding arthropods like *Aedes* mosquitoes. Predominantly transmitted by female *Aedes aegypti*, these viruses encode RNA genomes including dengue (DENV), Zika (ZIKV), chikungunya (CHIKV), yellow fever (YFV), and Ross River (RRV) viruses, etc. (1). *Aedes*-borne viruses are potentially deadly; they cause at least 40,000 deaths, each year (2). One of these viruses, DENV is endemic in over 141 countries, affects 390 million people, and claims 36,000 lives annually (3). As of right now, proper treatments for these viral diseases are unavailable. To combat this, different strategies focus on hosts, host-vector interaction, and the vectors themselves. From all these, vector control is a primary approach that involves chemical, environmental, and biological methods. Notably, one novel biological method is *Wolbachia*-based control, which may involve replacing wild-type mosquito populations with *Wolbachia*-infected variants. Additionally, *Wolbachia* can also inhibit viral proliferation in their host’s midguts, significantly reducing their ability to transmit viruses (4, 5). In the past two decades, establishing *Wolbachia*-infected *Ae. aegypti* population resistant to DENV, and investigating transgenic drivers for population replacement have substantially progressed (6, 7).

**Figure 1:**
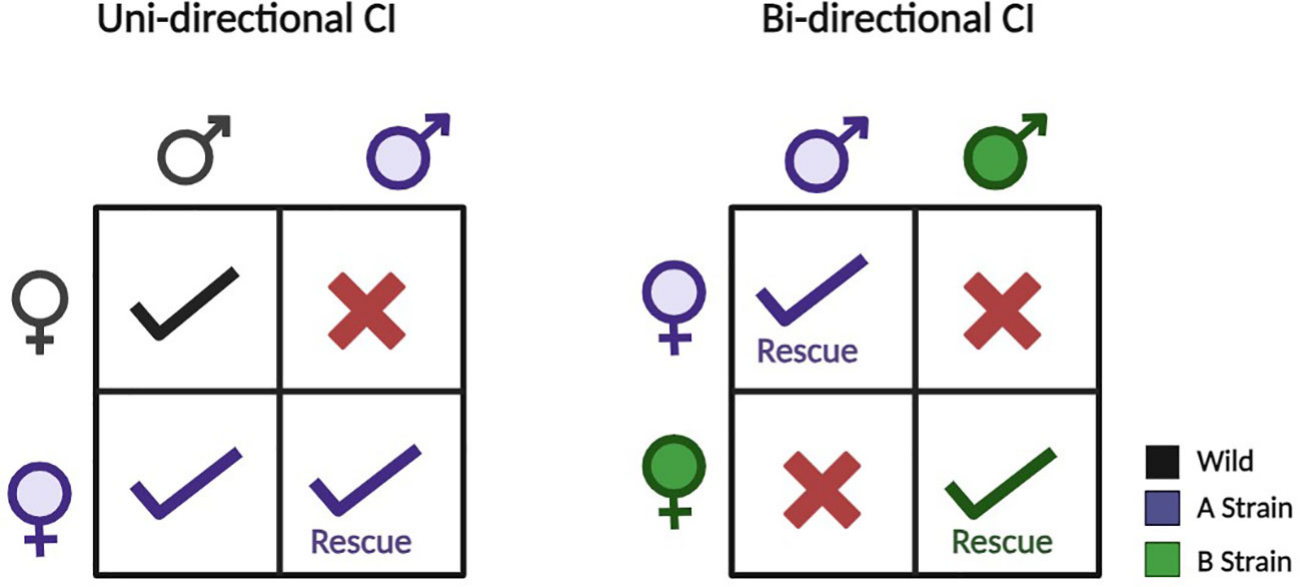
Uni- and bidirectional cytoplasmic incompatibility (CI) occur when crosses between infected and uninfected individuals lead to incompatible offspring. In unidirectional CI, only crosses with infected males and uninfected females are incompatible. In bidirectional CI, crosses between individuals infected with different CI-inducing strains are incompatible. Notably, unidirectional CI can sometimes occur between hosts infected with different bacterial strains. Figure created using BioRender.com.

Naturally, *Wolbachia* inhabits around 65% of all insect species (8) and in arthropods, this bacterium exhibits both mutualistic and parasitic interactions with its hosts (9). It provides multiple approaches to disease suppression such as by reducing vector population through incompatible males, affecting the fitness of the host, inhibiting pathogen transmission (10–13), affecting reproduction through male killing (14), feminization (15), parthenogenesis (16), and primarily, by cytoplasmic incompatibility (CI) (17, 18). In insects, CI occurs in two forms: (a) Unidirectional CI, where infected males can mate with only infected females with the same strain and cross with wild females resulting in embryo lethality and (b) Bidirectional CI, where males and females infected with different strains of *Wolbachia* cannot produce viable off-springs (**Figure 1**) (19). *Wolbachia*-infected females gain an evolutionary edge by mating with uninfected males, yielding viable offspring (20, 21). Manipulation of reproduction seems promising as it suggests that once a *Wolbachia* strain invades a target vector population through host reproductive alteration, ongoing management by health authorities might be minimized.

Anti-pathogenic effects of *Wolbachia* have been observed by many authors when it transfected non-native hosts (10, 22, 23). Although *Ae. aegypti* lacks natural association with *Wolbachia*, however, an uninfected *Ae. aegypti* laboratory population can quickly become infected when *Wolbachia*-infected females are introduced to the population (24). Hoffmann et al. (4) artificially introduced the *w*Mel strain of *Wolbachia* into *Ae. aegypti* mosquitoes. This strain induces CI, hampering breeding with *Wolbachia*-free mosquitoes. It was reported that it spreads quickly through mosquito populations, with little harm to the mosquitoes, and reaches high levels within just a few generations under semi-natural conditions. Most interestingly once a disease-blocking *Wolbachia* strain establishes itself in the target vector population, it can persist without further releases. It makes *Wolbachia* the best tool to inhibit arboviral transmission (25).

Investigating mosquito interactions with microorganisms, particularly with *Wolbachia*, reveals fascinating details about the defense mechanisms of the insects. Investigating mosquito interactions with microorganisms, particularly mosquitoes, like many insects, possess a vigorous innate immune system activated through pattern-recognition receptors (PRRs). In *Ae. aegypti*, both Toll and immune deficiency (IMD) signaling pathways induce antimicrobial peptides through transcription factors REL1 and REL2 (26). In transfected mosquito lines, the presence of *Wolbachia* activates the immune system, however, the precise function of these immune responses in establishing the symbiotic relationship between *Wolbachia* and the mosquitoes remains uncertain. Understanding how *Wolbachia* inhibits arboviruses is important for predicting factors that could affect both the viruses and mosquitoes. It will help to anticipate changes that might influence the effectiveness of *Wolbachia*-mediated inhibition. Gaining this understanding is essential to maximizing the *Wolbachia*-based control strategies’ durability and efficacy. This review article will go over how *Wolbachia* interferes with the way mosquito hosts, *Ae. aegypti*, interact with DENV, inhibits the entry and replication of viruses, reduces the amount of nutrients required for an arboviral infection, boosts immunity, produces reactive oxygen species (ROS), promotes cellular regeneration for a better midgut barrier, and controls genes involved in a range of cellular functions.

## 2 Defence systems of *Ae. aegypti* as a host

Pathogen-blocking mechanisms vary among host species, and a cellular process involved in pathogen blocking may not be generally applicable. It is commonly known that invertebrates, including *Ae. aegypti*, do not possess adaptive immunity. Mosquitoes employ defense mechanisms both within and outside their bodies to prevent pathogens and mainly rely on their innate immune system (27). It is now recognized that innate immunity in mosquitoes provides prompt defense against infections via humoral or cellular responses, which are typically brought on by the invasive microorganism. The cellular part involves special cells called hemocytes, while the humoral part includes various substances like PRR and anit-microbial peptides (AMPs). However, gene network analysis across insect species highlights strong connections between the pathways controlling the production of nutrients in the insect and the ability of viruses to replicate (28).

The genome of *Ae. aegypti*, known for its role as a disease vector, contains genes crucial for both viral infection and defense mechanisms. The most recent reference genome (AaegL5) reveals an expanded range of gene families, such as chemosensory receptors (related to the mosquito’s ability to sense chemicals), glutathione S-transferase (involved in detoxification processes), and C-type lectin (associated with immune responses), including specific genetic regions (chromosome 2) associated with viral susceptibility (29, 30). The presence of DENV, ZIKV, and CHIKV induces varying transcriptomic changes in *Ae. aegypti* (31). When these viruses infect *Ae. aegypti*, they trigger changes in the mosquito’s genetic activity in specific areas like cell structure, genetic processes, immune responses, stress reactions, and metabolic activities (32–34).

*Wolbachia* complicates the interaction between *Ae. aegypti* and arboviruses by disrupting the same molecular processes that are necessary for the viruses (33, 34–39). This interference causes cellular disturbances that harm the pathogen. Mosquitoes possess a natural defense mechanism against oxidative stress induced by blood meals. This defense involves activating antioxidants to protect their tissues. In DENV infection, mosquitoes produce ROS like mammalian cells but avoid the harmful effects associated with ROS accumulation (40). This unique ability is considered an evolutionary advantage, ensuring the successful transmission of the virus without compromising the mosquito’s health. DENV infection in mosquito cells (specifically C6/36 cells), causes endoplasmic reticulum (ER) stress by inducing the unfolded protein response, a cellular stress response mechanism. The chaperones GRP78/BiP and GRP94 are used as ER stress sensor genes, and their upregulation is observed against DENV in the cells of mosquitoes (41). Alterations in the mitochondrial membrane potential are linked to a noteworthy rise in GST (Glutathione S-Transferase) activity, suggesting the possibility of ER stress induction. Because mosquito cells have more GST activity, there may be less oxidative stress in the environment, which would facilitate viral propagation. Knocking down GST in DENV-infected cells elevates the concentration of superoxide dismutase, linking GST activity to oxidative stress regulation during DENV infection in mosquitoes (42, 43). GST also plays a significant role in minimizing cell death triggered by oxidative stress induced by DENV2 in mosquito cells (44). Additionally, eIF5A (an important protein involved in the complex process of protein synthesis) levels decrease during the aging of *Ae. aegypti* mosquitoes and its expression is upregulated in response to actively replicating DENV in the C6/36 cell line. It indicates a potential role for eIF5A in the cellular response to DENV infection (43, 45). Knowing all these cellular defense mechanisms in *Ae. aegypti* may help us to understand the mechanism behind *Wolbachia*-mediated control of DENV. Additionally, there is much evidence that the transinfection of various strains of *Wolbachia* into *Ae. aegypti* can prevent the spread of DENV (**Table 1**).

**Table 1:** Transaction of different *Wolbachia* strains into different cell lines results in DENV inhibition.

Wolbachia Strains	Cell line	DENV	Source
***w*AlbB**	**WB1,**	**Inhibition**	(24, 46)
	**Aag2 cell line**		(47)
	**C6/36 cells**		(48)
	***WB2* line**		(49)
	**Aag2**		(50)
	***w*RNase HI**		(51)
	***Ae. albopictus* cell line C6/36**		(22 **)**
	***Ae. aegypti* WB1**		(52 **)**
	***W-Aag2* cell line**		(46)
	**C6/36 cells**		(53)
	**NA**		(54)
	**C6/36 cells**		(55)
***w*Au and *w*AlbA**	**C6/36 cells**		(56)
***wMel***	***w*Mel-Aag2**		(50)
	**RML-12 cell line**		(4)
	**MGYP2 PGYP1**		(57)
	**MGYP2.out** **C6/36 cells**		(58)
	**Aag2**		(52 **)**
	**Aag2**		(59 **)**
	**Aag2**		(60)
	***Ae. aegypti* WB1**		(61)
	**RML-12 cell line**		(62)
	**C6/36 cells**		(63 **)**
	**No cell line mentioned**		(64–72)
***w*MelPop**	**PGYP1**		(73)
	***w*RNase HI**		(51)
	**Aag2**		(72)
	**PGYP1 *Ae. aegypti***		(52 **)**
			(57, 64)
***w*MelPop-CLA**	**C6/36.wMelPop-CLA line,**		(74 **)**
	**Aag2**		(72)
	**MGYP1.line PGYP1.out**		(10)
***w*MelPopCS**			(55)
***w*Pip**	***w*Pip-Aag2**		(50)

## 3 *Wolbachia*-*Aedes*-dengue association

**Figure 2:**
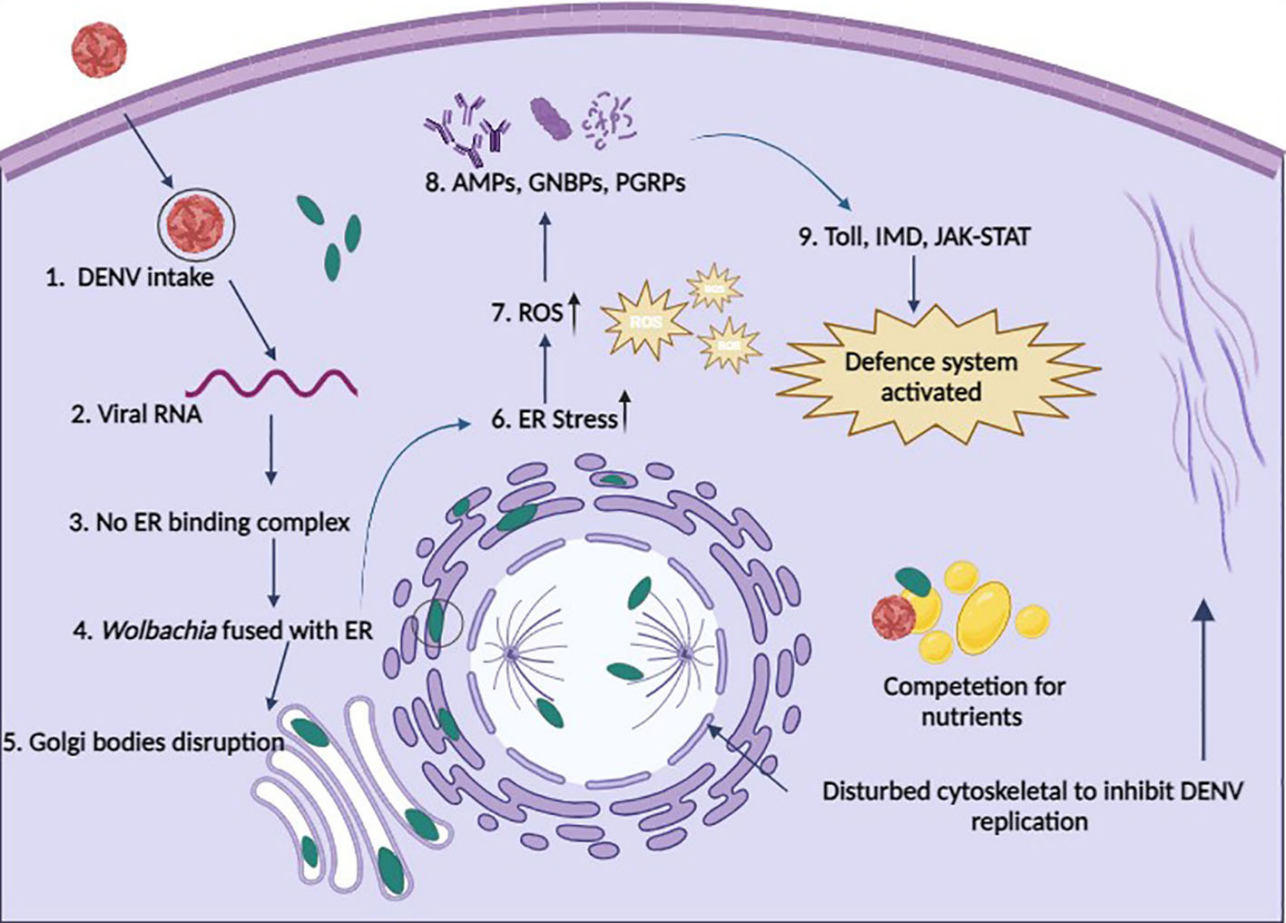
Possible defense systems of cells in the presence of *Wolbachia*. 1. DENV enters a *Wolbachia*-infected cell through endocytosis; 2. Viral RNA starts replication; 3. Replication of DENV is restricted because no binding complex forms on the ER membrane; 4. *Wolbachia* fused with the ER membrane and disturbs it; 5. No formation of Golgi vesicles due to disturbance of Golgi apparatus membrane by *Wolbachia*; 6. *Wolbachia* induces ER stress; 7. *Wolbachia* produces ROS to increase cellular stress; 8. Upregulation of AMPs, GNBPs, and PGRPs to boost immunity; 9. Immune pathways are activated to fight against pathogens (cells take *Wolbachia* as part of innate immunity); & *Wolbachia* also competes with DENV for nutrients and also disturbs the cytoskeleton to stop the movement of DENV and maturation.

The internal cellular structure of *Ae. aegypti* is required for arboviruses to successfully move through the stages of viral entrance, replication, assembly, and exit (75). This framework, referred to as the cytoskeleton, is made up of an actin filament and microtubule network. Arboviruses help build host cell structures for their survival, while *Wolbachia* does the opposite, weakening these structures to block the arboviral binding and entry (**Figure 2**) (9). In DENV-infected *Ae. aegypti*, genes for specific proteins such as dynein, vimentin, tubulin, actin, myosin, tropomyosin, and laminin are substantially expressed (76). Guo et al. (77) reported that actin and tubulin support DENV infection *in vitro*, while NS5 is associated with myosin in DENV infection (78). The introduction of the *w*AlbB strain appears to influence the cellular environment by reducing the levels of specific proteins associated with cell adhesion (dystroglycan) and cytoskeletal structure (beta-tubulin) in *Ae. aegypti* cells infected with DENV (79). This reveals a mechanism by which *Wolbachia* interferes with the virus’s development. This is a significant finding as it demonstrates how *Wolbachia* interferes with the early stages of arboviral infection (79).

## 4 How *Wolbachia* control DENV?

While *Wolbachia* is recognized for inhibiting certain viruses, its effectiveness is mainly observed against viruses with positive-sense or double-stranded RNA genomes (13). Its ability to inhibit negative-sense RNA viruses is less commonly reported. DENV, a positive-strand RNA virus, enters midgut cells following a blood meal (80). Many proteins, including replication factors, are produced when the viral RNA is translated into a polyprotein. Once DENV surpasses the midgut barrier, it can access other tissues like the fat body and the hemocytes. As soon as the virus enters the hemocoel, it can reproduce in the salivary gland cells and travel to the lumen of the glands. From there, the virus can be transmitted to a human host during subsequent mosquito blood-feeding. The exact mechanism behind *Wolbachia*-mediated blocking remains a mystery, primarily due to challenges in isolating the contributions of the three partners in the *Wolbachia*-*Aedes*-dengue association. The understanding of this process relies on observations of how these partners interact for a clear comprehension of the specific mechanisms involved (81). Some scientists suggest that *Wolbachia* may outcompete the virus for resources like lipids, enhance the mosquito’s immune system (82), and possibly this bacterium can lessen *Ae. aegypti*’s susceptibility to DENV (83). Additionally, there are many possible methods by which the transinfection of various strains of *Wolbachia* into *Ae. aegypti* can prevent the spread of disease. This section is all about how DENV affects the genes of *Ae. aegypti* and how the mosquito responds at the cellular level in the presence and absence of *Wolbachia*.

### 4.1 Competition for intracellular resources

Studies suggest that *Wolbachia*-induced metabolic changes in transinfected *Ae. aegypt*i may elucidate the pathogen-blocking mechanism (84–86). New studies suggest that instead of simply struggling over lipids, there’s a more complicated relationship where changes in lipids might work against each other. In one study by Koh et al., when DENV infection alone leads to an abundance of lipids *Wolbachia* and DENV both want the same things inside the cells of mosquitoes (87). *Wolbachia* relies on various host factors for replication, transmission, and manipulation of the host. It depends on host-derived membranes (88), altering their morphology, and affecting cholesterol/lipid metabolism (85). *Wolbachia* strategically localizes itself within vesicles closely associated with the endoplasmic reticulum, to gain access to the host cell’s lipid-rich environment (89). On the other hand, the DENV also disturbs the internal membranes of the cell to produce specific locations where the virus can multiply (90). By manipulating the cell’s fatty acid synthesis pathway, DENV effectively increases the production of lipids to facilitate its replication, while *Wolbachia* often triggers a response against pathogens in arthropods by competing for cholesterol and iron, necessary for their growth (46). *w*MelPop or *w*Mel infected cells of *Ae. aegypti* exhibit a significant reduction in total cholesterol (91) suggesting reliance on the host cell for lipid production due to lacking essential genes. This reduction in cholesterol impacts DENV replication, which also relies on cholesterol production. However, high *Wolbachia* abundance might consume excessive fatty acids, potentially disrupting normal cellular functions and virus replication. While the exact mechanism remains unclear, *Wolbachia* could be more resource-efficient than the virus, potentially enhancing mosquito immunity.

In *Ae. aegypti*, the *Wolbachia* strain or DENV disturbs cholesterol levels, resulting in increased cholesterol storage and localized lipid droplet accumulation (85). This dysregulation is marked by the upregulation of Niemann-Pick type C2, sterol carrier protein 2, and calnexin 99, associated with the downregulation of fatty acid synthase and LDL receptor proteins, indicative of compromised intracellular cholesterol transport. Specific lipids, like sphingomyelins and cardiolipins, are highly present in DENV3-infected mosquitoes but depleted when *w*Mel is present, suggesting an indirect antagonistic effect (87). In another study, the interaction involves elevated acyl-carnitine lipids during DENV infection but a significant reduction in *w*Mel-infected cells (92). Lowering acyl-carnitine increases *w*Mel density while adding this lipid to *w*Mel-infected cells boosts DENV. A recent study indicates that *w*Mel-transinfected *Ae. aegypti* suppresses DENV and ZIKV through the downregulation of the insulin receptor, however exact mechanisms need to be defined (93). In simple words, the virus may seek a lipid-rich environment for replication, which *Wolbachia* disrupts. However, understanding how *Wolbachia* downregulates the DENV is a matter of interest that is unclear.

### 4.2 Immune priming

To prevent arboviral transmission, *Wolbachia* employs two strategies. For starters, it competes for limited host cellular resources with arboviruses. Second, when transmitted to non-native hosts, it uses immune priming, which is a preactivation of the host’s immune system. This strengthens the arthropod’s resistance to arboviral infections. Signaling pathways such as IMD, Toll, and JAK-STAT initiate immune priming (94). Rances et al. (95) found that *Wolbachia* activates immunological genes linked to Toll pathways, melanization, and AMPs. The JAK-STAT pathway, known for regulating antiviral immunity, has been proven effective in preventing DENV infection in *Ae. aegypti* (96). *w*AlbB-transinfected *Ae. aegypti* upregulates Toll (GNBP1, SPZ3B, MYD88) and IMD (PRGP-LE, REL2) pathway genes, triggering the release of AMPs (e.g., cecropins, defensins) during arboviral infection (33, 34, 46). This immune-priming effect can be observed in mosquito larvae exposed to dormant dengue virus, resulting in protection against the virus in maturity (97).

#### 4.2.1 *Wolbachia* and Toll pathway

**Figure 3:**
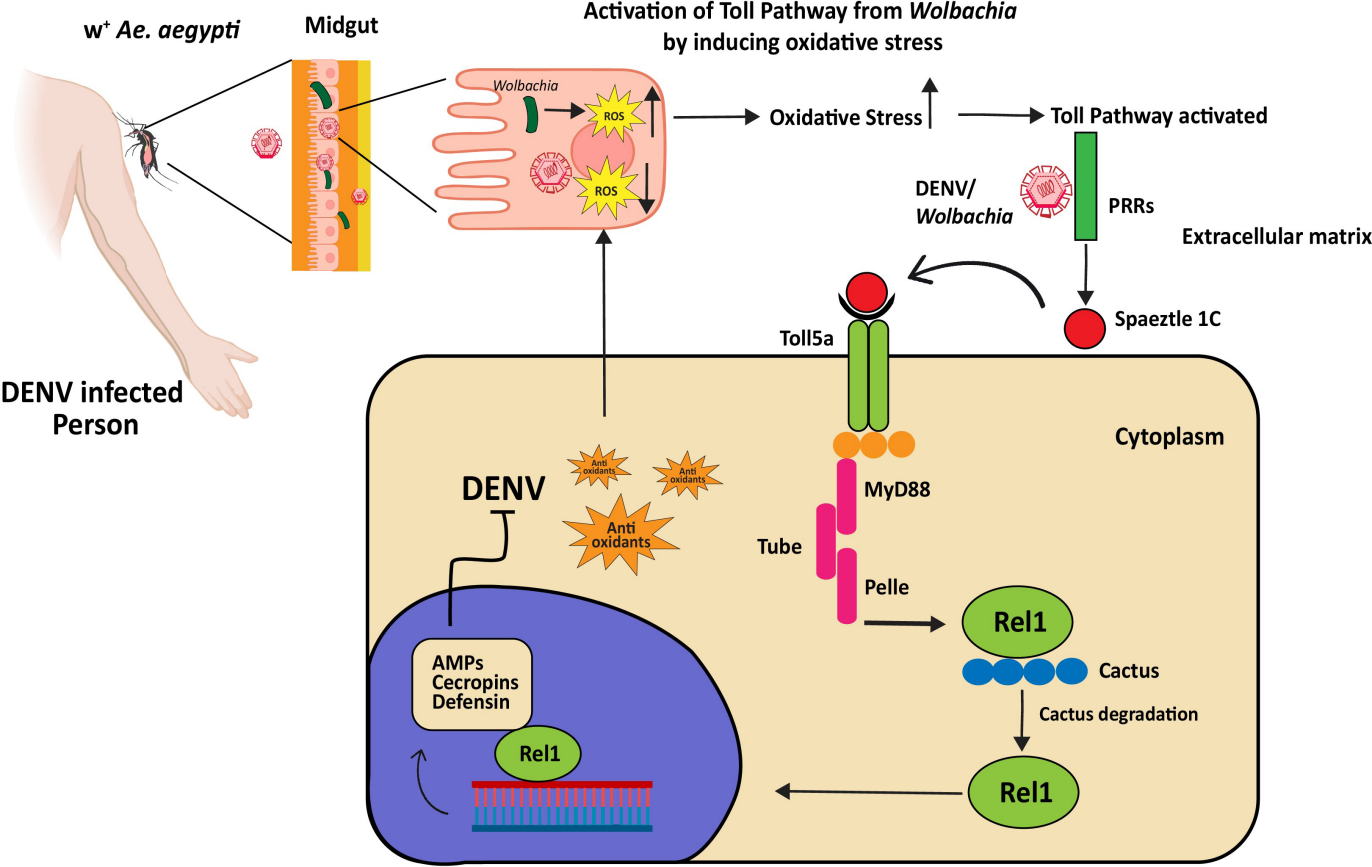
Dengue virus inhibition by *Wolbachia*-triggered Toll pathway activation in *Ae. aegypti*. *Wolbachia* produces ROS to favor its replication. To produce anti-oxidants to cope with oxidative stress, the Toll pathway is activated. The Toll pathway controls immune responses to *Wolbachia* and DENV through the systemic production of AMPs. PRRs recognize DENV or *Wolbachia*-associated molecular patterns and start maturation of spaetzle1C, it binds to Toll5a receptors and initiates the Toll pathway through adaptor proteins MyD88, Tube, and Pelle. The Cactus protein, a negative regulator of Rel1, is degraded by phosphorylation. Rel1 translocates into the nucleus and activates the transcription of genes encoding for AMPs, cecropin, and defending. These AMPs stop the replication of DENV (the exact mechanism is unknown).

Vector-virus interactions have been studied since the initial *Ae. aegypti* genome sequence was made public (98). *Ae. aegypti*’s defense against DENV infection is mediated by this pathway, as demonstrated by early transcriptome analysis in conjunction with functional assessments (99). Immune genes of the Toll pathway are upregulated in response to DENV-2 infection, indicating Myeloid Differentiation factor 88 (MYD88) is responsible for the high level of DENV and its essential role in controlling mosquito defense against DENV (33). *Wolbachia* activation of the Toll pathway induces the host release of ROS, leading to the synthesis of AMPs and antioxidants as shown in **Figure 3** (100). The silencing of the tissues of the midgut. In both the carcass and midgut tissue of *Ae. aegypti* infected with DENV, the AMP transcripts are highly marked (96). Furthermore, AMP gene expression is enhanced by the silencing of Cactus and Caspar (33). Viruses can modulate host arboviral susceptibility by downregulating AMP genes, as demonstrated in *in-vitro* and transcriptomic research on DENV-, ZIKV-, and CHIKV-infected mosquitoes (101). After infection, there’s a temporary increase in the expression of Spätzle (spz) and Rel1A, along with a transient rise in Cactus expression, which later decreases after 7 days (102). This upregulation indicates a robust immune response, with the pathway recognizing and combating the presence of the DENV in *Ae. aegypti*. When *Ae. aegypti* becomes infected with dengue, the Gram-negative binding proteins (GNBPs) may engage with virus particles or cellular debris, which could trigger immunological responses or directly neutralize virus particles, strengthening the mosquito’s defenses against DENV. Susceptibility has also been directly linked to several immune-related genes. Caicedo et al. (103) demonstrated that certain genes in *Ae. aegypti* significantly reduce the proliferation of DENV. These genes for specific proteins included Keratinocyte lectin *(AAEL009842*), GNBP *(AAEL009176)*, Cathepsin-b *(AAEL007585)*, and NPC2 *(AAEL015136)*. This demonstrates the significance of these genes and their role in the functioning of DENV infection. In mosquitoes, the midgut serves as a primary site for the replication of the virus and now it’s clear that the Toll pathway activation by RNAi-mediated depletion of Cactus suppresses viral infection in the mosquito midgut.

Bonizzoni et al. (104–106) found that extracellular PRR attaches to pathogen-derived ligands to initiate the Toll pathway. It triggers a proteolytic cascade that causes the Spätzle processing enzyme (SPE) to convert pro-Spätzle to Spätzle (107). Effector gene transcription is started when Spz binds to the transmembrane receptor Toll, triggering a cytoplasmic cascade that results in the nuclear translocation of the NF-kB transcription factor Rel1a. This pathway is noticeably downregulated in response to DENV infection specifically, certain variants of DENV-2, found in the 3’ untranslated region 3’UTR, inhibit the Toll pathway within mosquito salivary glands by producing subgenomic flaviviral RNA (108). However, there’s evidence suggesting that *Wolbachia* induces oxidative stress within the mosquito, and this stress, in turn, triggers the Toll pathway (100).

In the mosquito’s antiviral defense, multiple immune pathways are engaged, with each pathway showing specificity toward particular viruses. DENV virus activates Toll pathway genes, and increased expression of AMPs has been observed in these mosquitoes but their specific function in antiviral defense has yet to be fully understood. Pan et al. (100) suggested that the Toll pathway is responsible for expressing antioxidants and AMPs such as defensins and cecropins. Defensins were originally assumed to target enveloped viruses by breaking the viral envelope. Their extracellular antiviral impact is indicated by the fact that they are generated in the fat body and released into the hemolymph. *Wolbachia* infection activates defensins, including DEFA and CECA, to limit DENV proliferation, as demonstrated in DEF/CEC transgenic *Ae. aegypti* (109). Hence to fully understand mosquito antiviral defenses and their significance in the fight against infectious illnesses, more research is necessary.

#### 4.2.2 *Wolbachia* and IMD pathway

The IMD pathway is an important component of the insect defense system, particularly effective against gram-negative bacteria (110). Like the mammalian tumor necrosis factor signaling mechanism, the IMD pathway activates when membrane-bound PGRPs detect any pathogen. This triggers a signaling cascade involving the IMD protein, caspases, and kinases, ultimately leading to the activation of Rel2. It activates the transcription of AMPs and defense-related genes (111). When *D. melanogaster* gets infected by viruses, it activates the IMD pathway, which triggers the production of AMPs to fight off the invaders (112). Silencing key components of this pathway led to increased DENV titers in DENV-resistant mosquito strains, indicating its potential as an antiviral defense mechanism against this virus (113). Furthermore, *Wolbachia* activates this pathway, as a mechanism of defense in both natural host *Drosophila* and transinfected host *Ae. aegypti* mosquitoes (11, 22, 95).

**Figure 4:**
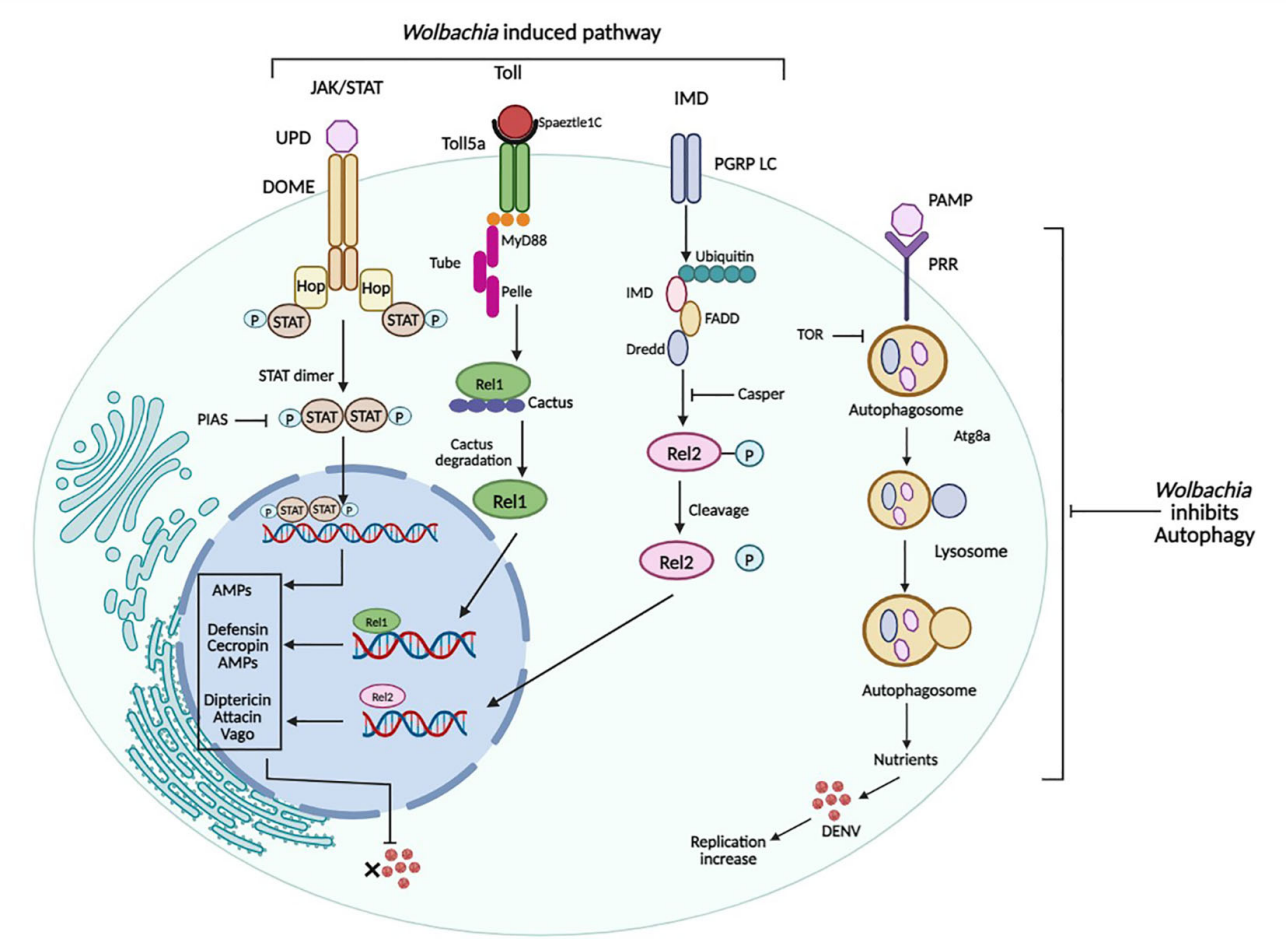
Immune pathways of *Ae. aegypti* and their regulation by *Wolbachia. Wolbachia* activates Toll, IMD, and JAK-STAT Pathway which in turn results in down-regulation of DENV. While *Wolbachia* inhibits autophagy, activated by DENV for its replication. Figure created in BioRender.com.

Ye et al. (57) reported that boosting the IMD pathway leads to higher *w*AlbB titers while silencing it leads to a decrease. The mosquito’s innate immune system can detect *w*AlbB through PGRP-LE, acting as a PRR and this triggers the activation of the IMD pathway. It is similar to how PGRP-LE functions as an intracellular sensor of Gram-negative bacteria in *Drosophila*, inducing the IMD pathway (110). Enhanced immunity boosts the expression of molecules that stimulate rather than inhibit *w*AlbB proliferation in *Ae. aegypti*, possibly because these AMPs lack specific targets on the *Wolbachia* cell membrane (46). Immune boosting may lead to increased *Wolbachia* density via the production of molecules that support *Wolbachia* replication, such as antioxidants as in the case of the Toll pathway (100). Increased production of AMPs by these antioxidants may indirectly benefit *Wolbachia* by removing susceptible microbial flora, allowing *Wolbachia* to occupy new niches. This suggests a positive feedback loop between host immune system activation and *Wolbachia* growth, aiding the establishment of *Wolbachia* symbiosis in transinfected *Ae. aegypti* lines. However, by activating the IMD pathway *Wolbachia* inhibits the replication of DENV in mosquitoes (**Figure 4**).

#### 4.2.3 *Wolbachia* and JAK-STAT pathway

The JAK-STAT pathway is crucial in *Ae. aegypti*’s defense against DENV, as suppressing it leads to increased virus replication in the mosquito midgut (114) and its activation, on the other hand, reduces virus replication. In *Ae. aegypti*, high JAK/STAT activation limits DENV replication, but the pathway’s effectors and the mechanisms behind JAK-STAT pathway-mediated antiviral effects are poorly understood. In *Ae. aegypti*, this pathway is activated by ligands such as Unpaired (Upd), which binds to the Dome receptor, leading to downstream signaling activation. Suppressing the Dome receptor or its JAK homolog HOP through RNA interference increases mosquito susceptibility to DENV infection while blocking the negative regulator, a protein inhibitor of activated STAT (PIAS), enhances DENV resistance (114). Two putative effector genes, DVRF1 and DVRF2, have been identified in *Ae. aegypti* as dengue virus restriction factors, but their functions are uncharacterized. The activation of various immunity-related genes by the JAK-STAT pathway suggests its role as a non-classical innate immune defense against DENV. Souza-Neto, Sim, & Dimopoulos (114) reported that *w*Mel induces the JAK/STAT pathway in *Ae. aegypti*, which controls DENV. However, the exact mechanism by which *Wolbachia* modulates the expression of the JAK/STAT pathways remains unclear.

#### 4.2.4 Transfected *Wolbachia* and mosquito immune responses

Naturally occurring *Wolbachia* does not have a major effect on vector competence in mosquito species. For example, *Ae. albopictus*’ *w*AlbB is unable to produce resistance against DENV in its native host (22). Bourtzis et al. (115) demonstrated that the AMP transcripts in *Ae*. *albopictus* is not substantially affected by *Wolbachia*. *Wolbachia*-infected mosquitoes exhibit resistance to diseases, which is probably the result of an increased host immune response that balances any potential negative consequences resulting from the recently acquired parasite. The key point here is that *Wolbachia*-induced immune factors activate pre-invasion, contrasting with pathogen-induced factors that activate post-invasion. Compared to the Toll pathway’s later activation by DENV*, Wolbachia* increases its activity before DENV invasion, allowing it to play a more significant role in clearing invasive viruses (33).

Many researchers explained the mechanisms through which *Wolbachia* activates the immune system of its host. In the case of *w*AlbB infected *Ae. aegypti*, Pan et al. (100) reported increased hydrogen peroxide (H_2_O_2_) levels and a significant increase in the expression of genes that encode NADPH oxidase (NOXM) and Dual Oxidase 2 (DUOX2) enzymes. These enzymes play a role in the production of ROS (116, 117). However, an upregulation of antioxidant genes in *Wolbachia*-infected mosquitoes implies the activation of mechanisms to neutralize ROS. Brennan et al. (35) also show ROS generation and antioxidant protein expression in *Wolbachia*-infected *Ae. albopictus* cell. ROS serve as messengers, activating NF-κB, a central regulator, to control immunity, inflammation, and cell survival (118).

Pan et al. (46) demonstrated that *Ae. aegypti*’s IMD and Toll pathways respond to *w*AlbB introduction, influencing infection levels. Activation increases *w*AlbB titer, while silencing reduces it, and elevated infection persists through maternal transmission. Remarkably, immune system amplification strongly promotes the synthesis of chemicals that actively promote *w*AlbB development in *Ae. aegypti* rather than merely failing to inhibit it. This is likely because there are no specific targets for AMPs in the *Wolbachia* cell membrane. The mosquito immune pathways trigger the effector molecules, such as *Wolbachia*-AMPs DEF and CEC, but surprisingly don’t impede *Wolbachia* growth. This immune system boost serves as a survival signal for the successful establishment of a novel *Wolbachia* symbiosis.

### 4.3 Autophagy

When cells face stress or starvation, autophagy helps get rid of damaged organelles and large protein aggregates. It can be used to degrade invasive bacteria, viruses, and parasites in addition to its function in recycling cell components during development (119). Autophagy is important for iron scavenging and cellular homeostasis and DENV induces and relies on autophagy for efficient viral replication in mammalian cells, despite its antiviral functions (120). DENV-induced autophagy, specifically targeting lipid droplets, alters cell metabolism, leading to the release of free fatty acids. Inhibition of this autophagy pathway inhibits DENV replication, suggesting that this pathway creates a favourable environment for viral replication by providing energy (121). Additionally, When *Wolbachia* is present, it manipulates the cell’s autophagy, impacting the replication of arboviruses. This interference limits the nutrients available for the viruses, making it harder for them to grow.

Activating autophagy decreases bacteria whereas suppressing it boosts bacterial populations in many organisms. *Wolbachia* levels are regulated by autophagy in a range of hosts, indicating the bacteria’s adaptation to resist autophagy and stay inside host cells. *Wolbachia* secretes a protein that manipulates the autophagy initiation pathway (122). Recent demonstrations show that ATG8a, a protein indicating autophagy activation, is abundant in *Brugia* tissues with high *Wolbachia* levels (123). Activation of the autophagy pathway triggered by *Wolbachia* infection is controlled by TOR–Atg1 signaling pathway genetic modification (123). Modification of TOR-Atg1 results in increased lysosomal production within the cell. *Wolbachia*-containing vacuoles can be bound by these lysosomes and eliminated. However, using a substance called 3-MA, autophagy could be inhibited which causes an increase in the quantity of *Wolbachia* in both animals and cells. *Wolbachia* most likely evolved anti-autophagy mechanisms to live and proliferate inside host cells. Furthermore, the APG5 is the most important gene of the autophagy (123). A recent finding revealed that there was no significant effect of *Wolbachia* infection on APG5 expression. Even though the load of DENV is high with the suppression of APG5, the *Wolbachia* presence does not alter the level of APG5. This indicates that in the presence of a *Wolbachia* infection autophagy is acting independently, but is probably a crucial factor in the *Ae. aegypti* against DENV infection.

### 4.4 miRNA-dependent immune pathways

The miRNA-dependent immune route is the fourth mechanism and it regulates numerous cellular functions, including transposon silencing, antiviral defense, differentiation, timing, cell division, and death, and is greatly aided by miRNAs. This pathway controls arboviral infection in diverse mosquito vectors by regulating arthropod host genes. Hussain et al. (36) concentrated on comprehending the impact of the *w*MelPop on cellular miRNAs in female mosquitoes. *aae-miR-2940-5p*, a mosquito-specific miRNA, is substantially increased in *w*MelPop-CLA-infected mosquitoes as opposed to uninfected mosquitoes. Mature *aae-miR-2940-5p* and pelo transcripts were found to co-localize by (124), suggesting the potential, in *Wolbachia*-containing cells, for aae-miR-2940-5p to downregulate the pelo transcripts. The immune response to viral infections consists of RNA interference (RNAi), a protective mechanism (125) that protects mosquitoes against DENV. In *Ae. aegypti* RNAi is the most important antiviral pathway, shown to reduce the proliferation of multiple viruses (DENV, chikungunya, and Sindbis viruses) but seems less crucial for blocking pathogens in naturally *Wolbachia*-infected insects (126). Activated by viral dsRNA cleavage, this pathway employs siRNAs to degrade viral ssRNA via cellular machinery. R2D2 and Dicer-2 are essential components of this pathway and if silenced mosquitoes are more susceptible to DENV (126). The RNase III domain of Dicer-2 cleaves the dsRNA after binding of Dicer-2-R2D2 complex to the viral dsRNA, for the formation of siRNA of 21– 23 nucleotides long. Now the siRNA will initiate the RNAi machinery by binding with RNA-induced silencing complex (RISC) which breaks the double-stranded RNA and unwinds one of the siRNA strands keeping the other for targeted degradation of single-stranded viral RNA with sequence complementary to the siRNA by the host endonuclease, Argonaute-2 (Ago2). Although RNAi activates against DENV, it doesn’t always stop the virus completely, emphasizing its role but limited effectiveness. To ensure the long-term survival of infected mosquitoes, it might just modify the replication of the virus to maintain chronic viral infection.

### 4.5 *Wolbachia* and specific immunity of *Ae. aegypti*

*Wolbachia* infects various tissues in the host, leading to significant impacts on host physiology (127). These effects extend to the cellular, individual, and population levels, affecting gene expression (33, 39), macromolecule availability (128), and fecundity, (129). The diversity of *Wolbachia’*s effects on the host highlights the complexity of this symbiotic relationship. *Wolbachia* combines reproductive manipulation, like cytoplasmic incompatibility, with mutualistic benefits, such as pathogen protection. The relationship spans a range between parasitism and mutualism. This dual impact makes *Wolbachia* a promising tool for controlling vector-borne diseases, using its influence on host reproduction and immune enhancement to reduce disease transmission. The mechanism through which *Wolbachia* provides antiviral protection is still a subject of ongoing research and discussion.

The example of DENV replication being seriously disrupted in the presence of *Wolbachia* is arguably the most thoroughly researched. Authors examine whether the Chromodomain helicase DNA binding proteins (CHD) may play a role in the interactions among *Wolbachia*, *Aedes*, and DENV. CHD proteins are a type of proteins classified within the ATP-dependent chromatin modifiers, specifically belonging to the SNF2 superfamily. Experimental evidence by (130) supports *AeCHD7*, a host component in *Ae. aegypti*, supporting DENV replication, while *Wolbachia’s* downregulation of it may inhibit DENV replication. Reduction in the expression levels of *AeCHD* genes is observed in mosquitoes infected with *Wolbachia*. *AeCHD7* promotes DENV replication, but *Wolbachia* reduces its expression in female *Ae. aegypti*, limiting the replication of DENV. This mechanism is only for female mosquitoes and not universally applicable across different hosts for *Wolbachia* to inhibit viral replication.

Asad et al. (131) discovered two vago proteins, *AeVago1* and *AeVago2*, in *Ae. aegypti*. Vago is a special antiviral protein found in insects. They investigated *AeVago1* production increased in *Wolbachia-*infected *Ae. aegypti.* Without changing the density of *Wolbachia*, *AeVago1* knockdown in *Wolbachia*-infected cells boosted DENV replication. Based on the data, it appears that *AeVago1* which *Wolbachia* induces in Aag2 cells, prevents DENV replication. Wu et al. (132) Lapidot et al. (133) have revealed the significance of the pelo protein for efficient viral replication, specifically for the *Drosophila* C virus and Tomato yellow leaf curl virus. Asad et al. (124) reported that *w*MelPop-CLA inhibits the pelo protein, and this inhibition might protect *Ae. aegypti* mosquitoes against DENV particles. *Ae. aegypti’s* tissues exhibit widespread expression of the pelo gene, with salivary gland expression being especially high but interestingly (134), but the presence of *Wolbachia* results in the suppression of pelo in various cell lines, salivary glands, muscles, and ovaries. In summary, the pelo protein promotes replication of DENV and on the other hand, *Wolbachia* inhibits the pelo protein in female *Ae. aegypti* mosquitoes, which may reduce DENV in these mosquitoes.

### 4.6 Unique miRNA expression in *Wolbachia* infection

Despite extensive research on *Wolbachia* biology, numerous unexplored mechanisms exist in its interactions with other organisms, suggesting manipulation of the host’s environment to ensure its survival. One such mechanism is the differential expression of mosquito cellular miRNAs due to *Wolbachia* infection (36). miRNAs function as post-transcriptional regulators, controlling multiple genes. In the presence of microbes, some miRNAs are dysregulated, while others are exclusively expressed, altering mosquito host responses as microbes persist within cells (135). Different *Wolbachia* strains have substantiated effects on *Ae. aegypti*’s miRNA profile such as in *Wolbachia*-infected mosquitoes, *w*Melpop-CLA induces exclusive miRNA expression, notably elevating *miR*-*2940*, which targets genes regulating *Wolbachia* density (36, 136). *miR-2940* enhances *w*Melpop-CLA replication by upregulating metalloprotease m41 ftsh and arginine methyl transferase 3 (AaArgM3) genes while inhibiting it reduces target gene expression and *Wolbachia* levels. Metalloprotease genes like m41 ftsh are upregulated by *miR-2940* in DENV-infected *Ae. aegypti* (106), and this miRNA is downregulated in WNV-infected cells (137). It suggests that *Wolbachia* may exploit host miRNAs to control essential host genes. On the other hand, *miR-2940* inhibits DNA methyltransferase (*AaDnmt2*) in *w*Melpop-CLA-infected mosquitoes (136). This gene is responsible for host defense and genome stability and is present abundantly in DENV-infected mosquitoes that are negative for *Wolbachia*. It indicates that *Wolbachia* creates a cellular environment incompatible with the virus by downregulating AaDnmt2 in *Ae. aegypti* (136).

Other miRNAs, can influence host autophagy and viral replication. For instance, *aae-miR-12* miRNAs, induced by *w*MelPop-CLA in Aag2 cells, can influence host autophagy and viral replication. For instance, *aae-miR-12* suppresses monocarboxylate transporter (MCT1) and DNA replication licensing factor (MCM6), potentially impacting autophagy pathways (138). MCT1’s involvement in autophagy is a matter of interest, which is exploited by DENV and ZIKV to evade host immune defenses (139). Further investigation is needed to determine if *Wolbachia*-produced miRNAs can modulate MCT1 activity and autophagy. *Wolbachia* infection also triggers the expression of *aae-miR-981*, resulting in the downregulation of importin b-4 in *w*MelPop-CLA-infected Aag2 cells (37). This reduction in importin b-4 activity inhibits the translocation of AGO1 to the nucleus. While the exact advantage of hindering AGO1 translocation for *Wolbachia’s* viral blocking remains unclear, importin b is known to assist in the nuclear migration of DENV and ZIKV non-structural proteins for optimal replication (140, 141). It suggests that the downregulation of importin b during *Wolbachia* infection may hinder viral transcription. Additionally *WsRNA-46*, a *Wolbachia*-derived miRNA in infected *Ae. aegypti*, promotes dynein expression, required for cellular transport and maintaining density in both *Wolbachia* and arboviruses, indicating an overlapping requirement for host cellular factors (142).

### 4.7 Fight for cytoskeletal components

Studies link *Wolbachia’s* pathogen-blocking effect to decreased viral load, but the mechanism and timing of interference in the virus life cycle are unclear. It was reported that *Wolbachia* interacts with the host cytoskeleton in two ways: by secreting effector molecules that bind to cytoskeletal structures to maintain optimal density and ensure transmission, and by regulating the expression of cytoskeletal proteins like dystroglycan and tubulin, crucial for arboviral infection (79). Arboviruses upregulate cytoskeletal structures for viral processes while the transinfected *w*AlbB strain in *Ae. aegypti* (Aag2) cells infected with DENV show downregulation of cytoskeletal membrane proteins, dystroglycan, and beta-tubulin. Silencing these cytoskeletal proteins inhibits DENV binding to Aag2 cells. This indicates *Wolbachia’s* direct involvement in hindering DENV binding and entry by targeting host cytoskeletal proteins utilized by the virus (106, 143).

### 4.8 Phenoloxidase cascade

The third mechanism disrupts arboviral transmission by triggering the phenoloxidase (PO) cascade. Melanin is produced by this cascade, which involves the enzyme phenoloxidase. Melanin exhibits antipathogenic properties when it accumulates around invasive pathogens and at wound sites (144, 145). The mosquito’s innate immune response to arboviruses depends on this process. Studies reveal that *Wolbachia* increases melanization in native and non-native arthropod vectors using phenoloxidase activities. Therefore, the phenoloxidase cascade that *Wolbachia* induces is probably a defense mechanism against different arboviral infections (146).

## 5 Discussion

This study analyzed different studies on the effect of *Wolbachia* on the immune system of hosts and offers an appealing mechanistic explanation for pathogen blocking. Recent field studies have demonstrated the effectiveness of *Wolbachia* in suppressing vector-borne disease transmission (4, 10, 13, 56, 72, 146, 147). These studies utilize three main strategies: (a) introducing *Wolbachia*-infected males to induce CI with uninfected females (4), (b) deploying *Wolbachia* strains that reduce mosquito fitness, such as by shortening lifespan, especially in regions with seasonal variation, (73) and (c) introducing *Wolbachia* strains that inhibit viral transmission by reducing vector competence (10, 13, 23, 72). These strategies, implemented in various countries including Australia, China, Indonesia, Brazil, and Vietnam, have shown promising results in controlling *Aedes*-borne viral infections.

*Wolbachia*-infected mosquitoes have been successfully used in over 14 countries, initially proven effective in Cairns, Australia in 2011 (4). In Brazil, after a resurgence of dengue in 1981, large-scale releases of *Wolbachia*-infected mosquitoes resulted in a notable 38% reduction in dengue and a 10% reduction in chikungunya (69). Yogyakarta, Indonesia also saw a significant 77% decrease in dengue transmission with *Wolbachia*-infected mosquito releases, accompanied by an 83% reduction in severe dengue cases (61, 148). In the USA, Myanmar, Malaysia, and China, the introduction of *w*AlbB-infected *Ae. aegypti* led to reduced human dengue incidence (54, 148, 149). Singapore’s release of the *w*AlbB strain in 2018 resulted in a 71-88% decrease in dengue cases (150). Cost-effectiveness analyses proposed implementing *Wolbachia* in high-risk urban areas of Vietnam, estimating significant reductions in dengue cases and associated economic benefits over 20 years (151). While field studies have demonstrated the effectiveness of *Wolbachia* in controlling mosquito-borne diseases, understanding the underlying mechanisms is crucial for optimizing its use.

Several mechanisms have been proposed to explain how *Wolbachia* inhibits virus replication in mosquitoes. Early in DENV infection, mosquitoes enhance innate immune genes, but as the infection progresses, it can suppress mosquito defenses, through the inhibition of immune-related genes (33, 76). However, the mosquitoes’ ability to compete with viruses can be modified by their microbiota. *Wolbachia*, a microbe, inhibits disease transmission by vectors, either by directly blocking virus transmission or reducing mosquito lifespan but the exact mechanism remains unclear due to *Wolbachia’s* inability to be cultured in a lab. Experimental evidence has repeatedly shown that *Wolbachia* is effective at preventing the replication of different flaviviruses, such as CHIKV, ZIKV, WNV, and DENV (10, 152, 153) with numerous studies demonstrating its significant inhibition of DENV replication (22, 136, 154). Transinfecting *Wolbachia* into *Ae. aegypti*, a vector not naturally hosting it, effectively inhibited DENV and CHIKV replication (155). Though much research has been done, the true mechanism or mechanisms through which *Wolbachia* inhibits viral reproduction in its host environment are still predominantly unknown.

*Wolbachia*, in general, boosts immune responses and increases resistance to viruses in mosquitoes (156). Additionally, mosquitoes infected with the *w*MelPop strain feed less as they age due to a bent proboscis, leading to reduced bite rates (157). Furthermore, the *w*Mel strain has been successfully transinfected into *Ae. aegypti*, inducing CI, ensuring high maternal transmission, and blocking the transmission of DENV (158). *Wolbachia* is believed to induce pathogen interference by activating the host’s innate immune system, particularly immune genes in the IMD and Toll pathways, such as REL1 and REL2 (10, 22, 23). Upregulation of immune effector genes is shown by *w*MelPop-CLA (10) and *w*AlbB (46) infected *Ae. aegypti*, by activation of IMD and Toll pathway. This activation increases the density of *Wolbachia* while turning off these pathways reduces it. The density increase may result from effector molecules that support *Wolbachia* replication and enhance the immune system. Such as the production of ROS that in turn initiates the Toll pathway (100) that is responsible for the inhibition of DENV. It demonstrates a positive feedback loop between the host immune system and *Wolbachia* density.

Another possibility for the observed effects of *Wolbachia* on DENV could be related to competition for essential nutrients. Cholesterol is recognized as a key fatty acid essential for the successful replication of DENV and *Wolbachia*. Substantive evidence suggests that *w*Mel competes with the DENV for limited sub-cellular fatty acid resources crucial for viral replication (4). Besides this when mosquitoes get infected with DENV there’s a natural defense system called autophagy that usually helps the virus grow. Chen and Smartt (159) discovered a surprising twist that this defense system might fight against the virus. DENV uses autophagy to help it grow. This special kind of autophagy focuses on lipid droplets and changes how the cell works. Interestingly, the *Wolbachia* hijack the cell’s internal process, to acquire nutrients it needs from the host. ATG8a, which indicates activation of autophagy, is found in large amounts in tissues of *the* host, where *Wolbachia* is also abundant (123). The reason is that *Wolbachia* relies on host cells for unsaturated fatty acids and may deplete these fatty acids, upsetting DENV replication. The hypothesis suggests that *Wolbachia’s* presence at high densities could inhibit viruses by competing for cholesterol, but experimental testing is needed for confirmation. Several aspects of immunity have been changed by *Wolbachia* in *Ae. aegypti* have been included in this review. Several other mechanisms are still not clear like, the connection between the lncRNA and the Toll pathway as *Wolbachia* uses lncRNA to activate the Toll pathway.

A radical effort is underway to combat dengue by using *Wolbachia* for long-term biological control. Recent studies are looking at immunity more completely. The lack of anti-dengue drugs highlights the importance of understanding how *Wolbachia* inhibits viral growth, which could inform new drug development. *Wolbachia* interferes with viruses by altering host factors necessary for viral replication. Future research should focus on identifying and characterizing these host factors, which could lead to novel strategies for controlling mosquito-borne diseases. Mosquito strains, carrying *Wolbachia* are currently bred and experimentally released in areas with a high public health burden of DENV transmission (59, 160). The ongoing *Wolbachia* releases offer a unique opportunity to predict the evolutionary impacts on the bacterium, virus, and mosquito host. This includes potential scenarios like the DENV partly evading transmission blockage and *Wolbachia* reducing its harmful effects on mosquitoes. These predictions aim to improve future forecasting and strategies. In the future, studies will try to understand how *Wolbachia* deals with the immune system, hormones, metabolism, and behavior of the host. To project the long-term stability of *Wolbachia–Ae. aegypti* mosquito system that controls mosquitoes and prevents dengue, we need to understand how *Wolbachia* and the host’s immunity work together.

## 6 Conclusions

Manipulation of mosquitoes’ innate immunity by *Wolbachia* to control diseases like malaria, dengue, chikungunya, and Zika is a rising strategy these days. However, its successful implementation relies on a thorough understanding of mosquito immunity and interactions with *Wolbachia and* viruses. This review concludes that *Ae. aegypti’*s innate immune response is essential to its ability to spread DENV, and using *Wolbachia* to boost immunity helps prevent DENV transmission. Even while the understanding of the host-*Wolbachia*-virus relationship has advanced significantly, there are still gaps in our understanding. Although the precise mechanism of antiviral defense is unknown. Determining the mechanism of *Wolbachia*-induced viral inhibition requires an understanding of mosquito innate immune responses in the presence of *Wolbachia*. This information is crucial for a major plan against arboviruses.

## Author contributions

IMus: Conceptualization, Data curation, Investigation, Writing – original draft. MS: Conceptualization, Data curation, Supervision, Validation, Writing – review & editing. IMun: Investigation, Writing – review & editing.

## References

[1] Guzman MG Harris E. Dengue. Lancet. (2015) 385:453–65. doi: 10.1016/S0140-6736(14)60572-9 25230594

[2] Bhatt S Gething PW Brady OJ Messina JP Farlow AW Moyes CL. The global distribution and burden of dengue. Nature. (2013) 496:504–7. doi: 10.1038/nature12060 PMC3651993 23563266

[3] World Mosquito Program. (2022). Available online at: www.worldmosquitoprogram.org.

[4] Hoffmann AA Montgomery BL Popovici J Iturbe-Ormaetxe I Johnson PH Muzzi F. Successful establishment of *Wolbachia* in *Aedes* populations to suppress dengue transmission. Nature. (2011) 476:454–7. doi: 10.1038/nature10356 21866160

[5] Joubert DA Walker T Carrington LB De Bruyne JT Kien DHT Hoang NLT. Establishment of a *Wolbachia* superinfection in *Aedes aEgypti* mosquitoes as a potential approach for future resistance management. PloS Pathog. (2016) 12:e1005434. doi: 10.1371/journal.ppat.1005434 26891349 PMC4758728

[6] Chen C-H Huang H Ward CM Su JT Schaeffer LV Guo M. A synthetic maternal-effect selfish genetic element drives population replacement in *Drosophila* . science. (2007) 316:597–600. doi: 10.1126/science.1138595 17395794

[7] Sinkins SP Gould F. Gene drive systems for insect disease vectors. Nat Rev Genet. (2006) 7:427–35. doi: 10.1038/nrg1870 16682981

[8] Werren JH Baldo L Clark ME. *Wolbachia*: master manipulators of invertebrate biology. Nat Rev Microbiol. (2008) 6:741–51. doi: 10.1038/nrmicro1969 18794912

[9] Reyes JIL Suzuki Y Carvajal T Muñoz MNM Watanabe K. Intracellular interactions between arboviruses and *Wolbachia* in *Aedes aEgypti* . Front Cell Infect Microbiol. (2021) 11:690087. doi: 10.3389/fcimb.2021.690087 34249780 PMC8261290

[10] Moreira LA Iturbe-Ormaetxe I Jeffery JA Lu G Pyke AT Hedges LM. A *Wolbachia* symbiont in *Aedes aEgypti* limits infection with dengue, Chikungunya, and *Plasmodium* . Cell. (2009) 139:1268–78. doi: 10.1016/j.cell.2009.11.042 20064373

[11] O’Connor L Plichart C Sang AC Brelsfoard CL Bossin HC Dobson SL. Open release of male mosquitoes infected with a *Wolbachia* biopesticide: field performance and infection containment. PloS Negl Trop Dis. (2012) 6:e1797. doi: 10.1371/journal.pntd.0001797 23166845 PMC3499408

[12] Rasic G Endersby NM Williams C Hoffmann AA. Using *Wolbachia*-based release for suppression of *Aedes* mosquitoes: insights from genetic data and population simulations. Ecol Appl. (2014) 24:1226–34. doi: 10.1890/13-1305.1 25154109

[13] Teixeira L Ferreira A Ashburner M. The bacterial symbiont *Wolbachia* induces resistance to RNA viral infections in *Drosophila melanogaster* . PloS Biol. (2008) 6:e2. doi: 10.1371/journal.pbio.1000002 PMC2605931 19222304

[14] Riparbelli MG Giordano R Ueyama M Callaini G. *Wolbachia*-mediated male killing is associated with defective chromatin remodeling. PloS One. (2012) 7:e30045. doi: 10.1371/journal.pone.0030045 22291901 PMC3264553

[15] Rousset F Bouchon D Pintureau B Juchault P Solignac M. *Wolbachia* endosymbionts responsible for various alterations of sexuality in arthropods. Proc R Soc London Ser B: Biol Sci. (1992) 250:91–8. doi: 10.1098/rspb.1992.0135 1361987

[16] Weeks AR Breeuwer JA. *Wolbachia*-induced parthenogenesis in a genus of phytophagous mites. Proc: Biol Sci. (2001) 268:2245–51. doi: 10.1098/rspb.2001.1797 PMC1088872 11674872

[17] Blagrove MS Arias-Goeta C Di Genua C Failloux A-B Sinkins SP. A *Wolbachia w*Mel transinfection in *Aedes albopictus* is not detrimental to host fitness and inhibits Chikungunya virus. PloS Negl Trop Dis. (2013) 7:e2152. doi: 10.1371/journal.pntd.0002152 23556030 PMC3610642

[18] Blagrove MS Arias-Goeta C Failloux AB Sinkins SP. *Wolbachia* strain *w*Mel induces cytoplasmic incompatibility and blocks dengue transmission in *Aedes albopictus* . Proc Natl Acad Sci United States America. (2012) 109:255–60. doi: 10.1073/pnas.1112021108 PMC3252941 22123944

[19] Stouthamer R Breeuwer JA Hurst GD. *Wolbachia pipientis*: microbial manipulator of arthropod reproduction. Annu Rev Microbiol. (1999) 53:71–102. doi: 10.1146/annurev.micro.53.1.71 10547686

[20] Turelli M Hoffmann AA. Rapid spread of an inherited incompatibility factor in California *Drosophila* . Nature. (1991) 353:440–2. doi: 10.1038/353440a0 1896086

[21] Yen JH Barr AR. The etiological agent of cytoplasmic incompatibility in *Culex pipiens* . J Invertebrate Pathol. (1973) 22:242–50. doi: 10.1016/0022-2011(73)90141-9 4206296

[22] Bian G Xu Y Lu P Xie Y Xi Z. The endosymbiotic bacterium *Wolbachia* induces resistance to dengue virus in *Aedes aEgypti* . PloS Pathog. (2010) 6:e1000833. doi: 10.1371/journal.ppat.1000833 20368968 PMC2848556

[23] Kambris Z Cook PE Phuc HK Sinkins SP. Immune activation by life-shortening *Wolbachia* and reduced filarial competence in mosquitoes. science. (2009) 326:134–6. doi: 10.1126/science.1177531 PMC2867033 19797660

[24] Xi Z Khoo CC Dobson SL. *Wolbachia* establishment and invasion in an *Aedes aEgypti* laboratory population. science. (2005) 310:326–8. doi: 10.1126/science.1117607 16224027

[25] Ross PA Turelli M Hoffmann AA. Evolutionary ecology of *wolbachia* releases for disease control. Annu Rev Genet. (2019) 53:93–116. doi: 10.1146/annurev-genet-112618-043609 31505135 PMC6944334

[26] Waterhouse RM Kriventseva EV Meister S Xi Z Alvarez KS Bartholomay LC. Evolutionary dynamics of immune-related genes and pathways in disease-vector mosquitoes. science. (2007) 316:1738–43. doi: 10.1126/science.1139862 PMC2042107 17588928

[27] Kumar A Srivastava P Sirisena P Dubey SK Kumar R Shrinet J. Mosquito innate immunity. Insects. (2018) 9:95. doi: 10.3390/insects9030095 30096752 PMC6165528

[28] Lindsey AR Bhattacharya T Hardy RW Newton IL. *Wolbachia* and virus alter the host transcriptome at the interface of nucleotide metabolism pathways. mBio. (2021) 12:e03472–03420. doi: 10.1128/mBio.03472-20 PMC7885120 33563832

[29] Adelman ZN Myles KM. The C-type lectin domain gene family in *Aedes aEgypti* and their role in arbovirus infection. Viruses. (2018) 10:367. doi: 10.3390/v10070367 30002303 PMC6070988

[30] Matthews BJ Dudchenko O Kingan S Koren S Antoshechkin I Crawford JE. Improved *Aedes aEgypt*i mosquito reference genome assembly enables biological discovery and vector control. BioRxiv. (2017) 563:501–7. doi: 10.1038/s41586-018-0692-z PMC6421076 30429615

[31] Mukherjee D Das S Begum F Mal S Ray U. The mosquito immune system and the life of dengue virus: what we know and do not know. Pathogens. (2019) 8:77. doi: 10.3390/pathogens8020077 31200426 PMC6631187

[32] Ramirez JL Dimopoulos G. The Toll immune signaling pathway control conserved anti-dengue defenses across diverse *Ae. aEgypt*i strains and against multiple dengue virus serotypes. Dev Comp Immunol. (2010) 34:625–9. doi: 10.1016/j.dci.2010.01.006 PMC2917001 20079370

[33] Xi Z Ramirez JL Dimopoulos G. The *Aedes aEgypti* toll pathway controls dengue virus infection. PloS Pathog. (2008) 4:e1000098. doi: 10.1371/journal.ppat.1000098 18604274 PMC2435278

[34] Zhao L Alto BW Jiang Y Yu F Zhang Y. Transcriptomic analysis of *aedes aEgypti* innate immune system in response to ingestion of chikungunya virus. Int J Mol Sci. (2019) 20:3133. doi: 10.3390/ijms20133133 31252518 PMC6651163

[35] Brennan LJ Keddie BA Braig HR Harris HL. The endosymbiont *Wolbachia pipientis* induces the expression of host antioxidant proteins in an *Aedes albopictus* cell line. PloS One. (2008) 3:e2083. doi: 10.1371/journal.pone.0002083 18461124 PMC2324199

[36] Hussain M Frentiu FD Moreira LA O’Neill SL Asgari S. *Wolbachia* uses host microRNAs to manipulate host gene expression and facilitate colonization of the dengue vector *Aedes aEgypti* . Proc Natl Acad Sci United States America. (2011) 108:9250–5. doi: 10.1073/pnas.1105469108 PMC3107320 21576469

[37] Hussain M O’Neill SL Asgari S. *Wolbachia* interferes with the intracellular distribution of Argonaute 1 in the dengue vector *Aedes aEgypti* by manipulating the host microRNAs. RNA Biol. (2013) 10:1868–75. doi: 10.4161/rna.27392 PMC3917989 24351659

[38] Lindsey AR Bhattacharya T Newton IL Hardy RW. Conflict in the intracellular lives of endosymbionts and viruses: a mechanistic look at *Wolbachia*-mediated pathogen-blocking. Viruses. (2018) 10:141. doi: 10.3390/v10040141 29561780 PMC5923435

[39] Zheng Y Wang JL Liu C Wang CP Walker T Wang YF. Differentially expressed profiles in the larval testes of *Wolbachia* infected and uninfected *Drosophila* . BMC Genomics. (2011) 12:595. doi: 10.1186/1471-2164-12-595 22145623 PMC3261232

[40] Birben E Sahiner UM Sackesen C Erzurum S Kalayci O. Oxidative stress and antioxidant defense. World Allergy Organ J. (2012) 5:9–19. doi: 10.1097/WOX.0b013e3182439613 23268465 PMC3488923

[41] Chen TH Chiang YH Hou JN Cheng CC Sofiyatun E Chiu CH. XBP1-mediated biP/GRP78 upregulation copes with oxidative stress in mosquito cells during dengue 2 virus infection. BioMed Res Int. (2017) 2017:3519158. doi: 10.1155/2017/3519158 29098151 PMC5642879

[42] Chen T-H Tang P Yang C-F Kao L-H Lo Y-P Chuang C-K. Antioxidant defense is one of the mechanisms by which mosquito cells survive dengue 2 viral infection. Virology. (2011) 410:410–7. doi: 10.1016/j.virol.2010.12.013 21216424

[43] Lin CC Yang CF Tu CH Huang CG Shih YT Chuang CK. A novel tetraspanin C189 upregulated in C6/36 mosquito cells following dengue 2 virus infection. Virus Res. (2007) 124:176–83. doi: 10.1016/j.virusres.2006.11.002 17156880

[44] Balakrishnan B Su S Wang K Tian R Chen M. Identification, expression, and regulation of an omega class glutathione S-transferase in rhopalosiphum padi (L.) (Hemiptera: aphididae) under insecticide stress. Front Physiol. (2018) 9:427. doi: 10.3389/fphys.2018.00427 29731722 PMC5920109

[45] Shih YT Yang CF Chen WJ. Upregulation of a novel eukaryotic translation initiation factor 5A (eIF5A) in dengue 2 virus-infected mosquito cells. Virol J. (2010) 7:214. doi: 10.1186/1743-422X-7-214 20819232 PMC2942825

[46] Pan X Pike A Joshi D Bian G McFadden MJ Lu P. The bacterium *Wolbachia* exploits host innate immunity to establish a symbiotic relationship with the dengue vector mosquito *Aedes aEgypti* . ISME J. (2018) 12:277–88. doi: 10.1038/ismej.2017.174 PMC5739022 29099491

[47] Lu P Bian G Pan X Xi Z. *Wolbachia* induces density-dependent inhibition to dengue virus in mosquito cells. PloS Negl Trop Dis. (2012) 6:e1754. doi: 10.1371/journal.pntd.0001754 22848774 PMC3404113

[48] Hugo LE Rašić G Maynard AJ Ambrose L Liddington C Thomas CJ. *Wolbachia w*AlbB inhibit dengue and Zika infection in the mosquito *Aedes aEgypti* with an Australian background. PloS Negl Trop Dis. (2022) 16:e0010786. doi: 10.1371/journal.pntd.0010786 36227923 PMC9562151

[49] Liu W-L Yu H-Y Chen Y-X Chen B-Y Leaw SN Lin C-H. Lab-scale characterization and semi-field trials of *Wolbachia* strain *w*AlbB in a Taiwan *Wolbachia* introgressed *Ae. aEgypti* strain. PloS Negl Trop Dis. (2022) 16:e0010084. doi: 10.1371/journal.pntd.0010084 35015769 PMC8752028

[50] Loterio RK Monson EA Templin R de Bruyne JT Flores HA Mackenzie JM. Antiviral *Wolbachia* strains associate with *Aedes aEgypti* endoplasmic reticulum membranes and induce lipid droplet formation to restrict dengue virus replication. mBio. (2023) 15:e02495–02423. doi: 10.1128/mbio.02495-23 PMC10865983 38132636

[51] Hussain M Zhang G Leitner M Hedges LM Asgari S. *Wolbachia* RNase HI contributes to virus blocking in the mosquito *Aedes aEgypti* . Iscience. (2023) 26:105836. doi: 10.1016/j.isci.2022.105836 36636344 PMC9830209

[52] Axford JK Ross PA Yeap HL Callahan AG Hoffmann AA. Fitness of *w*AlbB *Wolbachia* infection in *Aedes aEgypti*: parameter estimates in an outcrossed background and potential for population invasion. Am J Trop Med Hygiene. (2016) 94:507–16. doi: 10.4269/ajtmh.15-0608 PMC4775882 26711515

[53] Ahmad NA Mancini M-V Ant TH Martinez J Kamarul GM Nazni WA. *Wolbachia* strain *w*AlbB maintains high density and dengue inhibition following introduction into a field population of *Aedes aEgypti* . Philos Trans R Soc B. (2021) 376:20190809. doi: 10.1098/rstb.2019.0809 PMC7776933 33357050

[54] Nazni WA Hoffmann AA NoorAfizah A Cheong YL Mancini MV Golding N. Establishment of *Wolbachia* Strain *w*AlbB in Malaysian Populations of *Aedes aEgypti* for Dengue Control. Curr Biol. (2019) 29:4241–4248.e4245. doi: 10.1016/j.cub.2019.11.007 31761702 PMC6926472

[55] Flores HA Taneja de Bruyne J O’Donnell TB Tuyet Nhu V Thi Giang N Thi Xuan Trang H. Multiple *Wolbachia* strains provide comparative levels of protection against dengue virus infection in *Aedes aEgypti* . PloS Pathog. (2020) 16:e1008433. doi: 10.1371/journal.ppat.1008433 32282862 PMC7179939

[56] Ant TH Herd CS Geoghegan V Hoffmann AA Sinkins SP. The *Wolbachia* strain *w*Au provides highly efficient virus transmission blocking in *Aedes aEgypti* . PloS Pathog. (2018) 14:e1006815. doi: 10.1371/journal.ppat.1006815 29370307 PMC5784998

[57] Ye YH Woolfit M Rances E O’Neill SL McGraw EA. *Wolbachia*-associated bacterial protection in the mosquito *Aedes aEgypti* . PloS Negl Trop Dis. (2013) 7:e2362. doi: 10.1371/journal.pntd.0002362 23951381 PMC3738474

[58] Frentiu FD Zakir T Walker T Popovici J Pyke AT van den Hurk A. Limited dengue virus replication in field-collected *Aedes aEgypti* mosquitoes infected with *Wolbachia* . PloS Negl Trop Dis. (2014) 8:e2688. doi: 10.1371/journal.pntd.0002688 24587459 PMC3930499

[59] O’Neill SL Ryan PA Turley AP Wilson G Retzki K Iturbe-Ormaetxe I. Scaled deployment of *Wolbachia* to protect the community from dengue and other *Aedes* transmitted arboviruses. Gates Open Res. (2018) 2:36. doi: 10.12688/gatesopenres.12844.3 30596205 PMC6305154

[60] Indriani C Tantowijoyo W Rancès E Andari B Prabowo E Yusdi D. Reduced dengue incidence following deployments of *Wolbachia*-infected *Aedes aEgypti* in Yogyakarta, Indonesia: a quasi-experimental trial using controlled interrupted time series analysis. Gates Open Res. (2020) 4:50. doi: 10.12688/gatesopenres.13122.1 32803130 PMC7403856

[61] Utarini A Indriani C Ahmad RA Tantowijoyo W Arguni E Ansari MR. Efficacy of *Wolbachia*-infected mosquito deployments for the control of dengue. New Engl J Med. (2021) 384:2177–86. doi: 10.1056/NEJMoa2030243 PMC8103655 34107180

[62] Gu X Ross PA Rodriguez-Andres J Robinson KL Yang Q Lau MJ. A *w*Mel *Wolbachia* variant in *Aedes aEgypti* from field-collected *Drosophila melanogaster* with increased phenotypic stability under heat stress. Environ Microbiol. (2022) 24:2119–35. doi: 10.1111/1462-2920.15966 PMC9544352 35319146

[63] Pacidônio EC Caragata EP Alves DM Marques JT Moreira LA. The impact of *Wolbachia* infection on the rate of vertical transmission of dengue virus in Brazilian *Aedes aEgypti* . Parasites Vectors. (2017) 10:1–6. doi: 10.1186/s13071-017-2236-z 28623959 PMC5474007

[64] Ferguson NM Hue Kien DT Clapham H Aguas R Trung VT Bich Chau TN. Modeling the impact on virus transmission of *Wolbachia*-mediated blocking of dengue virus infection of *Aedes aEgypti* . Sci Trans Med. (2015) 7:279ra237–279ra237. doi: 10.1126/scitranslmed.3010370 PMC4390297 25787763

[65] Carrington LB Tran BCN Le NTH Luong TTH Nguyen TT Nguyen PT. Field-and clinically derived estimates of *Wolbachia*-mediated blocking of dengue virus transmission potential in *Aedes aEgypti* mosquitoes. Proc Natl Acad Sci. (2018) 115:361–6. doi: 10.1073/pnas.1715788115 PMC5777059 29279375

[66] Ryan PA Turley AP Wilson G Hurst TP Retzki K Brown-Kenyon J. Establishment of *w*Mel *Wolbachia* in *Aedes aEgypti* mosquitoes and reduction of local dengue transmission in Cairns and surrounding locations in northern Queensland, Australia. Gates Open Res. (2019) 3:1547. doi: 10.12688/gatesopenres.13061.2 31667465 PMC6801363

[67] Ford SA Allen SL Ohm JR Sigle LT Sebastian A Albert I. Selection on *Aedes aEgypti* alters *Wolbachia*-mediated dengue virus blocking and fitness. Nat Microbiol. (2019) 4:1832–9. doi: 10.1038/s41564-019-0533-3 PMC6990461 31451771

[68] Pinto SB Riback TI Sylvestre G Costa G Peixoto J Dias FB. Effectiveness of *Wolbachia*-infected mosquito deployments in reducing the incidence of dengue and other *Aedes*-borne diseases in Niterói, Brazil: A quasi-experimental study. PloS Negl Trop Dis. (2021) 15:e0009556. doi: 10.1371/journal.pntd.0009556 34252106 PMC8297942

[69] Ribeiro dos Santos G Durovni B Saraceni V Souza Riback TI Pinto SB Anders KL. Estimating the impact of the *w*Mel release program on dengue and chikungunya incidence in Rio de Janeiro, Brazil. Medrxiv. (2022) 22:P1587–1595. doi: 10.1016/S1473-3099(22)00436-4 PMC9630156 36182679

[70] Velez ID Tanamas SK Arbelaez MP Kutcher SC Duque SL Uribe A. Reduced dengue incidence following city-wide *w*Mel *Wolbachia* mosquito releases throughout three Colombian cities: Interrupted time series analysis and a prospective case-control study. PloS Negl Trop Dis. (2023) 17:e0011713. doi: 10.1371/journal.pntd.0011713 38032857 PMC10688673

[71] Hue KDT Goncalves DS Vi TT Long VT Nhu VTT Giang NT. *Wolbachia w*Mel-mediated effects on dengue virus vertical transmission from *Aedes aEgypti* to their offspring. Parasites Vectors. (2023) 308:308. doi: 10.1186/s13071-023-05921-y PMC10472731 37653429

[72] Walker T Johnson P Moreira L Iturbe-Ormaetxe I Frentiu F McMeniman C. The *w*Mel *Wolbachia* strain blocks dengue and invades caged *Aedes aEgypti* populations. Nature. (2011) 476:450–3. doi: 10.1038/nature10355 21866159

[73] McMeniman CJ Lane RV Cass BN Fong AW Sidhu M Wang Y-F. Stable introduction of a life-shortening *Wolbachia* infection into the mosquito *Aedes aEgypti* . science. (2009) 323:141–4. doi: 10.1126/science.1165326 19119237

[74] Frentiu FD Robinson J Young PR McGraw EA O’Neill SL. *Wolbachia*-mediated resistance to dengue virus infection and death at the cellular level. PloS One. (2010) 5:e13398. doi: 10.1371/journal.pone.0013398 20976219 PMC2955527

[75] Foo KY Chee H-Y. Interaction between flavivirus and cytoskeleton during virus replication. BioMed Res Int. (2015) 2015:427814. doi: 10.1155/2015/427814 26347881 PMC4546964

[76] Sim S Dimopoulos G. Dengue virus inhibits immune responses in *Aedes aEgypt*i cells. PloS One. (2010) 5:e10678. doi: 10.1371/journal.pone.0010678 20502529 PMC2872661

[77] Guo X Xu Y Bian G Pike AD Xie Y Xi Z. Response of the mosquito protein interaction network to dengue infection. BMC Genomics. (2010) 11:380. doi: 10.1186/1471-2164-11-380 20553610 PMC3091628

[78] Paingankar MS Gokhale MD Deobagkar DN. Dengue-2-virus-interacting polypeptides involved in mosquito cell infection. Arch Virol. (2010) 155:1453–61. doi: 10.1007/s00705-010-0728-7 20571839

[79] Lu P Sun Q Fu P Li K Liang X Xi Z. *Wolbachia* inhibits binding of dengue and Zika viruses to mosquito cells. Fronters Microbiol. (2020) 11:1750. doi: 10.3389/fmicb.2020.01750 PMC7417768 32849379

[80] Salazar MI Richardson JH Sanchez-Vargas I Olson KE Beaty BJ. Dengue virus type 2: replication and tropisms in orally infected *Aedes aEgypti* mosquitoes. BMC Microbiol. (2007) 7:9. doi: 10.1186/1471-2180-7-9 17263893 PMC1797809

[81] Johnson KN. The impact of *wolbachia* on virus infection in mosquitoes. Viruses. (2015) 7:5705–17. doi: 10.3390/v7112903 PMC4664976 26556361

[82] Frayer K. *Wolbachia* goes to work in the war on mosquitoes. Nature. (2021) 598:S33.

[83] Edenborough KM Flores HA Simmons CP Fraser JE. Using *Wolbachia* to eliminate dengue: Will the virus fight back? J Virol. (2021) 95:e02203–02220. doi: 10.1128/JVI.02203-20 PMC8253515 33853965

[84] Cui Y Liu P Mooney BP Franz AW. Quantitative proteomic analysis of chikungunya virus-infected *Aedes aEgypti* reveals proteome modulations indicative of persistent infection. J Proteome Res. (2020) 19:2443–56. doi: 10.1021/acs.jproteome.0c00173 PMC7419016 32375005

[85] Geoghegan V Stainton K Rainey SM Ant TH Dowle AA Larson T. Perturbed cholesterol and vesicular trafficking associated with dengue blocking in *Wolbachia*-infected *Aedes aEgypti* cells. Nat Commun. (2017) 8:526. doi: 10.1038/s41467-017-00610-8 28904344 PMC5597582

[86] Leier HC Weinstein JB Kyle JE Lee J-Y Bramer LM Stratton KG. A global lipid map defines a network essential for Zika virus replication. Nat Commun. (2020) 11:3652. doi: 10.1038/s41467-020-17433-9 32694525 PMC7374707

[87] Koh C Islam MN Ye YH Chotiwan N Graham B Belisle JT. Dengue virus dominates lipid metabolism modulations in *Wolbachia*-coinfected *Aedes aEgypti* . Commun Biol. (2020) 3:518. doi: 10.1038/s42003-020-01254-z 32948809 PMC7501868

[88] White PM Serbus LR Debec A Codina A Bray W Guichet A. Reliance of *wolbachia* on high rates of host proteolysis revealed by a genome-wide RNAi screen of *drosophila* cells. Genetics. (2017) 205:1473–88. doi: 10.1534/genetics.116.198903 PMC5378107 28159754

[89] Cho K-O Kim G-W Lee O-K. *Wolbachia* bacteria reside in host Golgi-related vesicles whose position is regulated by polarity proteins. PloS One. (2011) 6:e22703. doi: 10.1371/journal.pone.0022703 21829485 PMC3145749

[90] Perera R Riley C Isaac G Hopf-Jannasch AS Moore RJ Weitz KW. Dengue virus infection perturbs lipid homeostasis in infected mosquito cells. PloS Pathog. (2012) 8:e1002584. doi: 10.1371/journal.ppat.1002584 22457619 PMC3310792

[91] Caragata EP Rancès E O’Neill SL McGraw EA. Competition for amino acids between *Wolbachia* and the mosquito host, *Aedes aEgypti* . Microb Ecol. (2014) 67:205–18. doi: 10.1007/s00248-013-0339-4 24337107

[92] Manokaran G Flores HA Dickson CT Narayana VK Kanojia K Dayalan S. Modulation of acyl-carnitines, the broad mechanism behind *Wolbachia*-mediated inhibition of medically important flaviviruses in *Aedes aEgypti* . Proc Natl Acad Sci United States America. (2020) 117:24475–83. doi: 10.1073/pnas.1914814117 PMC7533870 32913052

[93] Haqshenas G Terradas G Paradkar PN Duchemin JB McGraw EA Doerig C. A role for the insulin receptor in the suppression of dengue virus and zika virus in *wolbachia*-infected mosquito cells. Cell Rep. (2019) 26:529–535 e523. doi: 10.1016/j.celrep.2018.12.068 30650347

[94] Kamtchum-Tatuene J Makepeace BL Benjamin L Baylis M Solomon T. The potential role of *Wolbachia* in controlling the transmission of emerging human arboviral infections. Curr Opin Infect Dis. (2017) 30:108–16. doi: 10.1097/QCO.0000000000000342 PMC5325245 27849636

[95] Rances E Ye YH Woolfit M McGraw EA O’Neill SL. The relative importance of innate immune priming in *Wolbachia*-mediated dengue interference. PloS Pathog. (2012) 8:e1002548. doi: 10.1371/journal.ppat.1002548 22383881 PMC3285598

[96] Jupatanakul N Sim S Anglero-Rodriguez YI Souza-Neto J Das S Poti KE. Engineered *aedes aEgypti* JAK/STAT pathway-mediated immunity to dengue virus. PloS Negl Trop Dis. (2017) 11:e0005187. doi: 10.1371/journal.pntd.0005187 28081143 PMC5230736

[97] Vargas V Cime-Castillo J Lanz-Mendoza H. Immune priming with inactive dengue virus during the larval stage of *Aedes aEgypti* protects against the infection in adult mosquitoes. Sci Rep. (2020) 10:6723. doi: 10.1038/s41598-020-63402-z 32317699 PMC7174395

[98] Nene V Wortman JR Lawson D Haas B Kodira C Tu ZJ. Genome sequence of Aedes aEgypti, a major arbovirus vector. science. (2007) 316:1718–23. doi: 10.1126/science.1138878 PMC2868357 17510324

[99] Weaver SC Vasilakis N. Molecular evolution of dengue viruses: contributions of phylogenetics to understanding the history and epidemiology of the preeminent arboviral disease. Infect Genet Evol. (2009) 9:523–40. doi: 10.1016/j.meegid.2009.02.003 PMC3609037 19460319

[100] Pan X Zhou G Wu J Bian G Lu P Raikhel AS. *Wolbachia* induces reactive oxygen species (ROS)-dependent activation of the Toll pathway to control dengue virus in the mosquito *Aedes aEgypti* . Proc Natl Acad Sci United States America. (2012) 109:E23–31. doi: 10.1073/pnas.1116932108 PMC3252928 22123956

[101] Carvalho-Leandro D Ayres C Guedes D Suesdek L Melo-Santos M Oliveira CF. Immune transcript variations among *Aedes aEgypti* populations with distinct susceptibility to dengue virus serotype 2. Acta Tropica. (2012) 124:113–9. doi: 10.1016/j.actatropica.2012.07.006 22877626

[102] Souza-Neto JA Powell JR Bonizzoni M. *Aedes aEgypti* vector competence studies: A review. Infect Genet Evol. (2019) 67:191–209. doi: 10.1016/j.meegid.2018.11.009 30465912 PMC8135908

[103] Caicedo PA Serrato IM Sim S Dimopoulos G Coatsworth H Lowenberger C. Immune response-related genes associated to blocking midgut dengue virus infection in *Aedes aEgypti* strains that differ in susceptibility. Insect Sci. (2019) 26:635–48. doi: 10.1111/1744-7917.12573 29389079

[104] Bonizzoni M Dunn WA Campbell CL Olson KE Marinotti O James AA. Complex modulation of the *Aedes aEgypti* transcriptome in response to dengue virus infection. PloS One. (2012) 7:e50512. doi: 10.1371/journal.pone.0050512 23209765 PMC3507784

[105] Luplertlop N Surasombatpattana P Patramool S Dumas E Wasinpiyamongkol L Saune L. Induction of a peptide with activity against a broad spectrum of pathogens in the *Aedes aEgypti* salivary gland, following Infection with Dengue Virus. PloS Pathog. (2011) 7:e1001252. doi: 10.1371/journal.ppat.1001252 21249175 PMC3020927

[106] Sim S Ramirez JL Dimopoulos G. Dengue virus infection of the *Aedes aEgypti* salivary gland and chemosensory apparatus induces genes that modulate infection and blood-feeding behavior. PloS Pathog. (2012) 8:e1002631. doi: 10.1371/journal.ppat.1002631 22479185 PMC3315490

[107] DeLotto Y DeLotto R. Proteolytic processing of the *Drosophila* Spatzle protein by easter generates a dimeric NGF-like molecule with ventralising activity. Mech Dev. (1998) 72:141–8. doi: 10.1016/s0925-4773(98)00024-0 9533958

[108] Pompon J Manuel M Ng GK Wong B Shan C Manokaran G. Dengue subgenomic flaviviral RNA disrupts immunity in mosquito salivary glands to increase virus transmission. PloS Pathog. (2017) 13:e1006535. doi: 10.1371/journal.ppat.1006535 28753642 PMC5555716

[109] Kokoza V Ahmed A Woon Shin S Okafor N Zou Z Raikhel AS. Blocking of *Plasmodium* transmission by cooperative action of Cecropin A and Defensin A in transgenic *Aedes aEgypti* mosquitoes. Proc Natl Acad Sci United States America. (2010) 107:8111–6. doi: 10.1073/pnas.1003056107 PMC2889521 20385844

[110] Kaneko T Silverman N. Bacterial recognition and signalling by the *Drosophila* IMD pathway. Cell Microbiol. (2005) 7:461–9. doi: 10.1111/j.1462-5822.2005.00504.x 15760446

[111] Stöven S Silverman N Junell A Hedengren-Olcott M Erturk D Engström Y. Caspase-mediated processing of the *Drosophila* NF-κB factor Relish. Proc Natl Acad Sci. (2003) 100:5991–6. doi: 10.1073/pnas.1035902100 PMC156314 12732719

[112] Avadhanula V Weasner BP Hardy GG Kumar JP Hardy RW. A novel system for the launch of alphavirus RNA synthesis reveals a role for the Imd pathway in arthropod antiviral response. PloS Pathog. (2009) 5:e1000582. doi: 10.1371/journal.ppat.1000582 19763182 PMC2738967

[113] Xi Z Gavotte L Xie Y Dobson SL. Genome-wide analysis of the interaction between the endosymbiotic bacterium *Wolbachia* and its *Drosophila* host. BMC Genomics. (2008) 9:1–12. doi: 10.1186/1471-2164-9-1 18171476 PMC2253531

[114] Souza-Neto JA Sim S Dimopoulos G. An evolutionary conserved function of the JAK-STAT pathway in anti-dengue defense. Proc Natl Acad Sci United States America. (2009) 106:17841–6. doi: 10.1073/pnas.0905006106 PMC2764916 19805194

[115] Bourtzis K Pettigrew M O’Neill SL. *Wolbachia* neither induces nor suppresses transcripts encoding antimicrobial peptides. Insect Mol Biol. (2000) 9:635–9. doi: 10.1046/j.1365-2583.2000.00224.x 11122472

[116] Kawahara T Quinn MT Lambeth JD. Molecular evolution of the reactive oxygen-generating NADPH oxidase (Nox/Duox) family of enzymes. BMC Ecol Evol. (2007) 7:109. doi: 10.1186/1471-2148-7-109 PMC1940245 17612411

[117] Kumar S Molina-Cruz A Gupta L Rodrigues J Barillas-Mury C. A peroxidase/dual oxidase system modulates midgut epithelial immunity in *Anopheles Gambiae* . science. (2010) 327:1644–8. doi: 10.1126/science.1184008 PMC3510679 20223948

[118] Morgan MJ Liu ZG. Crosstalk of reactive oxygen species and NF-kappaB signaling. Cell Res. (2011) 21:103–15. doi: 10.1038/cr.2010.178 PMC3193400 21187859

[119] Merkling SH van Rij RP. Beyond RNAi: antiviral defense strategies in *Drosophila* and mosquito. J Insect Physiol. (2013) 59:159–70. doi: 10.1016/j.jinsphys.2012.07.004 22824741

[120] Lee YR Lei HY Liu MT Wang JR Chen SH Jiang-Shieh YF. Autophagic machinery activated by dengue virus enhances virus replication. Virology. (2008) 374:240–8. doi: 10.1016/j.virol.2008.02.016 PMC7103294 18353420

[121] Heaton NS Randall G. Dengue virus-induced autophagy regulates lipid metabolism. Cell Host Microbe. (2010) 8:422–32. doi: 10.1016/j.chom.2010.10.006 PMC3026642 21075353

[122] Niu H Xiong Q Yamamoto A Hayashi-Nishino M Rikihisa Y. Autophagosomes induced by a bacterial Beclin 1 binding protein facilitate obligatory intracellular infection. Proc Natl Acad Sci. (2012) 109:20800–7. doi: 10.1073/pnas.1218674109 PMC3529060 23197835

[123] Voronin D Cook DA Steven A Taylor MJ. Autophagy regulates *Wolbachia* populations across diverse symbiotic associations. Proc Natl Acad Sci United States America. (2012) 109:E1638–1646. doi: 10.1073/pnas.1203519109 PMC3382551 22645363

[124] Asad S Hussain M Hugo L Osei-Amo S Zhang G Watterson D. Suppression of the pelo protein by *Wolbachia* and its effect on dengue virus in *Aedes aEgypti* . PloS Negl Trop Dis. (2018) 12:e0006405. doi: 10.1371/journal.pntd.0006405 29641562 PMC5912784

[125] Blair CD. Mosquito RNAi is the major innate immune pathway controlling arbovirus infection and transmission. Future Microbiol. (2011) 6:265–77. doi: 10.2217/fmb.11.11 PMC3126673 21449839

[126] Sanchez-Vargas I Scott JC Poole-Smith BK Franz AW Barbosa-Solomieu V Wilusz J. Dengue virus type 2 infections of *Aedes aEgypti* are modulated by the mosquito’s RNA interference pathway. PloS Pathog. (2009) 5:e1000299. doi: 10.1371/journal.ppat.1000299 19214215 PMC2633610

[127] Dobson SL Bourtzis K Braig HR Jones BF Zhou W Rousset F. *Wolbachia* infections are distributed throughout insect somatic and germ line tissues. Insect Biochem Mol Biol. (1999) 29:153–60. doi: 10.1016/s0965-1748(98)00119-2 10196738

[128] Molloy JC Sommer U Viant MR Sinkins SP. *Wolbachia* modulates lipid metabolism in *aedes albopictus* mosquito cells. Appl Environ Microbiol. (2016) 82:3109–20. doi: 10.1128/AEM.00275-16 PMC4959074 26994075

[129] Stouthamer R Luck R. Influence of microbe-associated parthenogenesis on the fecundity of *Trichogramma deion* and *T. pretiosum* . Entomol Experimentalis Applicata. (1993) 67:183–92. doi: 10.1111/j.1570-7458.1993.tb01667.x

[130] Asad S Hall-Mendelin S Asgari S. Downregulation of *Aedes aEgypti* chromodomain helicase DNA binding protein 7/Kismet by *Wolbachia* and its effect on dengue virus replication. Scencei Rep. (2016) 6:36850. doi: 10.1038/srep36850 PMC5101808 27827425

[131] Asad S Parry R Asgari S. Upregulation of *Aedes aEgypti* Vago1 by *Wolbachia* and its effect on dengue virus replication. Insect Biochem Mol Biol. (2018) 92:45–52. doi: 10.1016/j.ibmb.2017.11.008 29157676

[132] Lapidot M Karniel U Gelbart D Fogel D Evenor D Kutsher Y. A novel route controlling begomovirus resistance by the messenger RNA surveillance factor pelota. PloS Genet. (2015) 11:e1005538. doi: 10.1371/journal.pgen.1005538 26448569 PMC4598160

[133] Wu X He W-T Tian S Meng D Li Y Chen W. Pelo is required for high efficiency viral replication. PloS Pathog. (2014) 10:e1004034. doi: 10.1371/journal.ppat.1004034 24722736 PMC3983054

[134] Shamsadin R Adham IM von Beust G Engel W. Molecular cloning, expression and chromosome location of the human pelota gene PELO. Cytogenet Cell Genet. (2000) 90:75–8. doi: 10.1159/000015667 11060452

[135] Feng X Zhou S Wang J Hu W. microRNA profiles and functions in mosquitoes. PloS Negl Trop Dis. (2018) 12:e0006463. doi: 10.1371/journal.pntd.0006463 29718912 PMC5951587

[136] Zhang G Hussain M O’Neill SL Asgari S. *Wolbachia* uses a host microRNA to regulate transcripts of a methyltransferase, contributing to dengue virus inhibition in *Aedes aEgypti* . Proc Natl Acad Sci United States America. (2013) 110:10276–81. doi: 10.1073/pnas.1303603110 PMC3690878 23733960

[137] Slonchak A Hussain M Torres S Asgari S Khromykh AA. Expression of mosquito microRNA Aae-miR-2940-5p is downregulated in response to West Nile virus infection to restrict viral replication. J Virol. (2014) 88:8457–67. doi: 10.1128/JVI.00317-14 PMC4135961 24829359

[138] Osei-Amo S Hussain M O’Neill SL Asgari S. *Wolbachia*-induced aae-miR-12 miRNA negatively regulates the expression of *MCT1* and MCM6 genes in *Wolbachia*-infected mosquito cell line. PloS One. (2012) 7:e50049. doi: 10.1371/journal.pone.0050049 23166816 PMC3500346

[139] Velentzas PD Zhang L Das G Chang T-K Nelson C Kobertz WR. The proton-coupled monocarboxylate transporter hermes is necessary for autophagy during cell death. Dev Cell. (2018) 47:281–293. e284. doi: 10.1016/j.devcel.2018.09.015 30318245 PMC6219939

[140] Ji W Luo G. Zika virus NS5 nuclear accumulation is protective of protein degradation and is required for viral RNA replication. Virology. (2020) 541:124–35. doi: 10.1016/j.virol.2019.10.010 32056710

[141] Pryor MJ Rawlinson SM Butcher RE Barton CL Waterhouse TA Vasudevan SG. Nuclear localization of dengue virus nonstructural protein 5 Through Its Importin α/β–recognized nuclear localization sequences is integral to viral infection. Traffic (Copenhagen Denmark). (2007) 8:795–807. doi: 10.1111/j.1600-0854.2007.00579.x 17537211

[142] Baldridge GD Baldridge AS Witthuhn BA Higgins L Markowski TW Fallon AM. Proteomic profiling of a robust *Wolbachia* infection in an *Aedes albopictus* mosquito cell line. Mol Microbiol. (2014) 94:537–56. doi: 10.1111/mmi.12768 PMC4213348 25155417

[143] Sim S Jupatanakul N Ramirez JL Kang S Romero-Vivas CM Mohammed H. Transcriptomic profiling of diverse *Aedes aEgypti* strains reveals increased basal-level immune activation in dengue virus-refractory populations and identifies novel virus-vector molecular interactions. PloS Negl Trop Dis. (2013) 7:e2295. doi: 10.1371/journal.pntd.0002295 23861987 PMC3701703

[144] Rodriguez-Andres J Rani S Varjak M Chase-Topping ME Beck MH Ferguson MC. Phenoloxidase activity acts as a mosquito innate immune response against infection with Semliki Forest virus. PloS Pathog. (2012) 8:e1002977. doi: 10.1371/journal.ppat.1002977 23144608 PMC3493465

[145] Thomas P Kenny N Eyles D Moreira LA O’Neill SL Asgari S. Infection with the *w*Mel and *w*MelPop strains of *Wolbachia* leads to higher levels of melanization in the hemolymph of *Drosophila melanogaster*, *Drosophila simulans* and *Aedes aEgypti* . Dev Comp Immunol. (2011) 35:360–5. doi: 10.1016/j.dci.2010.11.007 21075139

[146] Rainey SM Shah P Kohl A Dietrich I. Understanding the *Wolbachia*-mediated inhibition of arboviruses in mosquitoes: progress and challenges. J Gen Virol. (2014) 95:517–30. doi: 10.1099/vir.0.057422-0 24343914

[147] Hoffmann AA Ross PA Rasic G. *Wolbachia* strains for disease control: ecological and evolutionary considerations. Evolutionary Applications. (2015) 8(8):751–68. 10.1111/eva.12286 PMC4561566 26366194

[148] Indriani C Tanamas SK Khasanah U Ansari MR Rubangi Tantowijoyo W. Impact of randomised wmel *Wolbachia* deployments on notified dengue cases and insecticide fogging for dengue control in Yogyakarta City. Glob Health Action. (2023) 16(1):2166650. 36700745 10.1080/16549716.2023.2166650 PMC9894080

[149] Crawford JE Clarke DW Criswell V Desnoyer M Cornel D Deegan B. Efficient production of male *Wolbachia*-infected Aedes aegypti mosquitoes enables large-scale suppression of wild populations. Nature Biotechnology. (2020) 38(4):482–92. 10.1038/s41587-020-0471-x 32265562

[150] Zheng X Zhang D Li Y Yang C Wu Y Liang X. Incompatible and sterile insect techniques combined eliminate mosquitoes. Nature. (2019) 572(7767):56–61. 10.1038/s41586-019-1407-9 31316207

[151] Tantowijoyo W Tanamas SK Nurhayati I Setyawan S Budiwati N Fitriana I. Aedes aegypti abundance and insecticide resistance profiles in the applying *Wolbachia* to eliminate dengue trial. PLoS Neglected Tropical Diseases. (2022) 16(4):e0010284. 35442957 10.1371/journal.pntd.0010284 PMC9060332

[152] Dutra HL Rocha MN Dias FB Mansur SB Caragata EP Moreira LA. *Wolbachia* blocks currently circulating zika virus isolates in Brazilian *aedes aEgypt*i mosquitoes. Cell Host Microbe. (2016) 19:771–4. doi: 10.1016/j.chom.2016.04.021 PMC4906366 27156023

[153] Hussain M Lu G Torres S Edmonds JH Kay BH Khromykh AA. Effect of *Wolbachia* on replication of West Nile virus in a mosquito cell line and adult mosquitoes. J Virol. (2013) 87:851–8. doi: 10.1128/JVI.01837-12 PMC3554047 23115298

[154] Hughes H Britton NF. Modelling the use of *Wolbachia* to control dengue fever transmission. Bull Math Biol. (2013) 75:796–818. doi: 10.1007/s11538-013-9835-4 23535905

[155] McMeniman CJ Lane AM Fong AW Voronin DA Iturbe-Ormaetxe I Yamada R. Host adaptation of a *Wolbachia* strain after long-term serial passage in mosquito cell lines. Appl Environ Microbiol. (2008) 74:6963–9. doi: 10.1128/AEM.01038-08 PMC2583474 18836024

[156] Zhang D Wang Y He K Yang Q Gong M Ji M. *Wolbachia* limits pathogen infections through induction of host innate immune responses. PloS One. (2020) 15:e0226736. doi: 10.1371/journal.pone.0226736 32078642 PMC7032688

[157] Turley AP Moreira LA O’Neill SL McGraw EA. *Wolbachia* infection reduces blood-feeding success in the dengue fever mosquito, *Aedes aEgypti* . PloS Negl Trop Dis. (2009) 3:e516. doi: 10.1371/journal.pntd.0000516 19753103 PMC2734393

[158] Walker T Johnson PH Moreira LA Iturbe-Ormaetxe I Frentiu FD McMeniman CJ. The wMel *Wolbachia* strain blocks dengue and invades caged Aedes aegypti populations. Nature. (2011) 476(7361):450–3. 10.1038/nature10355 21866159

[159] Chen TY Smartt CT. Activation of the autophagy pathway decreases dengue virus infection in *Aedes aEgypti* cells. Parasites Vectors. (2021) 14:551. doi: 10.1186/s13071-021-05066-w 34702321 PMC8549150

[160] Carrington LB Tran BCN Le NTH Luong TTH Nguyen TT Nguyen PT. Field- and clinically derived estimates of *Wolbachia*-mediated blocking of dengue virus transmission potential in Aedes aegypti mosquitoes. Proceedings of the National Academy of Sciences of the United States of America. (2018) 115(2):361–6. 10.1073/pnas.1715788115 PMC5777059 29279375

